# Quantitative profiling of the vaginal microbiota improves resolution of the microbiota-immune axis

**DOI:** 10.1186/s40168-025-02039-4

**Published:** 2025-02-04

**Authors:** Eric Armstrong, Rachel Liu, James Pollock, Sanja Huibner, Suji Udayakumar, Erastus Irungu, Pauline Ngurukiri, Peter Muthoga, Wendy Adhiambo, Sergey Yegorov, Joshua Kimani, Tara Beattie, Bryan Coburn, Rupert Kaul

**Affiliations:** 1 Toronto General Hospital Research Institute, University Health Network, Toronto, Canada; 2 Department of Medicine, University of Toronto, Toronto, Canada; 3 Department of Immunology, University of Toronto, Toronto, Canada; 4 Partners for Health and Development in Africa (PHDA), Nairobi, Kenya; 5 Department of Biochemistry and Biomedical Sciences, McMaster University, Hamilton, Canada; 6 Department of Global Health and Development, London School of Hygiene and Tropical Medicine, London, UK

## Abstract

**Background:**

The composition of the vaginal microbiota is closely linked to adverse sexual and reproductive health outcomes, due in part to effects on genital immunology. Compositional approaches such as metagenomic sequencing provide a snapshot of all bacteria in a sample and have become the standard for characterizing the vaginal microbiota, but only provide microbial relative abundances. We hypothesized that the addition of absolute abundance data would provide a more complete picture of host-microbe interactions in the female genital tract.

**Results:**

We analyzed cervicovaginal secretions from 196 female sex workers in Kenya and found that bacterial load was elevated among women with diverse, bacterial vaginosis (BV)-type microbiota and lower among women with *Lactobacillus* predominance. Bacterial load was also positively associated with proinflammatory cytokines, such as IL-1α, and negatively associated with chemokines, such as IP-10. The associations between bacterial load and immune factors differed across bacterial community states, but *L. crispatus* predominance was the only microbial community where higher bacterial load was not associated with higher proinflammatory cytokines. Total vaginal bacterial load was also a stronger predictor of the genital immune environment than BV by Nugent score, the current clinical standard, in the Kenya-based cohort and in a Uganda-based confirmatory cohort.

**Conclusions:**

Our results suggest that total vaginal bacterial load is at least as strong a predictor of the genital immune milieu as current BV clinical diagnostic tools, supporting exploration of the vaginal bacterial load as a predictor of adverse reproductive and sexual health outcomes.

Video Abstract

**Supplementary Information:**

The online version contains supplementary material available at 10.1186/s40168-025-02039-4.

## Introduction

The vaginal microbiota is an important determinant of female reproductive and sexual health [1]. A state of microbial diversity, commonly known as bacterial vaginosis (BV), is associated with adverse sexual and reproductive health outcomes such as elevated risk of HIV acquisition [2] and preterm birth [3], whereas vaginal predominance by *Lactobacillus* species, particularly *Lactobacillus crispatus*, is protective [4]. These adverse outcomes are believed to be mediated, at least in part, by the induction of a pro-inflammatory genital immune milieu and the associated breakdown of epithelial barrier integrity [5, 6]. Traditionally, BV has either been defined clinically, based on the presence of symptoms and clinical findings (i.e., Amsel BV) [7], or microbiologically, based on the proportions of lactobacilli and diverse Gram-negative and curved Gram variable bacilli on a microscopic smear (i.e., Nugent BV) [8]. However, these methods do not capture the broad range of vaginal microbiota composition that is found in women with BV. In research settings, most studies exploring the vaginal microbiota now employ molecular techniques such as 16S rRNA gene sequencing or metagenomic sequencing to characterize the proportional abundance of all bacteria or organisms, respectively. These methods allow for the characterization of nearly all microbes in a sample, but they only measure relative, rather than absolute, bacterial abundances [9]. An important limitation of relative abundance data is that it can obscure causal relationships between microbes and host processes and disease when they are determined by the absolute abundance of microbial community members [10]. Given these limitations, elucidation of the mechanism(s) by which microbial dynamics influence host immunology and clinical outcomes might be enhanced by the use of absolute rather than relative bacterial abundances. Indeed, this has been shown in the gut, where patterns of bacterial co-occurrence differ considerably based on whether relative or absolute quantification is used, and where associations between bacterial community composition and disease state are due to absolute rather than relative bacterial load [11, 12]. This approach has been less explored in the vaginal microbiota, and we hypothesized that absolute bacterial quantification would enhance our understanding of the vaginal microbiota-immune axis.

## Materials and methods

### Study design

Female sex workers in Nairobi, Kenya provided informed, written consent and were recruited through the Sex Worker Outreach Program (SWOP) clinics. Briefly, consenting participants who were not pregnant or breastfeeding and did not have a chronic illness (diabetes, rheumatoid arthritis, asthma, TB infection, or chirotherapy in the past 6 months) were enrolled in the study and completed HIV testing (rapid test and HIV GNA GeneXpert, Cephoid), STI testing and a behavioral-biological survey, which primarily included questions on adverse childhood events, mental health, and violence. Additional details on the behavioral-biological survey have been described previously [13]. Participants provided blood, urine, cervicovaginal secretions (SoftCup collections; Evofem, San Diego, CA, USA), and vaginal swabs for immune and microbial studies. Blood plasma was tested for antibodies against syphilis and HSV-2, and urine samples were used to screen for pregnancy, *Chlamydia trachomatis* (GeneXpert Assay, Cephoid), and *Neisseria gonorrhoeae* (GeneXpert Assay, Cephoid). Self-collected vaginal swabs were used for Nugent scoring and tested for *Trichomonas vaginalis* (OSOM Trichomonas Rapid Test, SEKISUI Diagnostics, LLC)*.* Cervicovaginal secretions were analyzed for soluble immune factors and microbiota characterization. This subset of participants was selected from a larger cohort of 1003 female sex workers under the age of 45 who were randomly selected across SWOP clinics. A subset of participants from this cohort were selected for detailed vaginal microbiota characterization in the present analysis. Exclusion criteria for this sub-study included the presence of an STI (*Chlamydia trachomatis*, *Neisseria gonorrhoeae*, and *Trichomonas vaginalis),* forced sex in the last 7 days, more than 10 clients in the past 7 days, and a history of female genital mutilation. Participants who tested positive for herpes simplex virus 2 (HSV-2) or syphilis were not excluded. HSV-2 positivity has been shown not to be inflammatory with regard to vaginal soluble immune factors [14] and syphilis is unlikely to be inflammatory unless primary or secondary [15], which were not observed in this cohort. From the larger cohort of eligible participants, we randomly selected 196 participants for inclusion in this sub-study at a ratio of 2:1 for participants with either intermediate microbiota or BV (defined as a Nugent score of 4–10) vs. participants with normal microbiota (defined as a Nugent score of 1–3). Study design and other methods for the Uganda-based confirmatory cohort can be found in the Supplemental methods.

### Sample processing

Cervicovaginal secretions (CVS) were collected by SoftCup, diluted tenfold in sterile PBS, and centrifuged at 1730 g for 10 min. Supernatants were extracted, and the remaining pellets were resuspended in 500 μL of sterile PBS. Both the supernatants and pellet were frozen at –80 °C and transported to the University of Toronto for analysis.

### Soluble immune factor measurement

Cervicovaginal secretion supernatants were thawed and re-centrifuged at 2000 rpm for 5 min. The following soluble immune factors were assayed: IL-1α, IL-1β, IL-6, IL-8, MCP-1, TNF-α, MIP-1α, MIP-1β, MIP-3α, IP-10, MIG, soluble E-cadherin (sE-cad), and MMP-9 in duplicate by Multiplex MSD according to the manufacturer’s instructions (Meso Scale Discovery, Rockville, MD, USA). Soluble E-cadherin and MMP-9 are used as markers of epithelial disruption [16, 17]. CVS supernatants were plated at 25 μL per well and a standard curve was used to determine the lower and upper limit of detection, and the concentration of each analyte (pg/mL). The upper limit of quantification (ULOQ) and lower limit of quantification (LLOQ) were determined by the lowest value and highest value, respectively, across all plates analyzed. Samples that were below the LLOQ were designated the LLOQ value, and samples above the ULOQ were assigned the ULOQ value.

### DNA extraction and 16S rRNA gene sequencing

DNA was isolated from 250 μL of the CVS pellet using the DNEasy PowerSoil Pro Kit (Qiagen), according to the manufacturer’s instructions. DNA was eluted in 50 μL of the Qiagen elution buffer. The V4 hypervariable region of the 16S rRNA gene was amplified using uniquely barcoded 515F (forward) and 806R (reverse) sequencing primers to allow for multiplexing [18]. Amplification reactions were performed using 12.5 μL of KAPA2G Robust HotStart ReadyMix (KAPA Biosystems), 1.5 μL of 10 μM forward and reverse primers, 7.5 μL of sterile water and 2 μL of DNA. The V4 region was amplified by cycling the reaction at 95 °C for 3 min, × 18 cycles of 95 °C for 15 s, 50 °C for 15 s, and 72 °C for 15 s, followed by a 5-min 72 °C extension. All amplification reactions were done in duplicate to reduce amplification bias, pooled, and checked on a 1% agarose TBE gel. Pooled duplicates were quantified using PicoGreen and combined by even concentrations. The library was then purified using Ampure XP beads and loaded onto the Illumina MiSeq for sequencing, according to manufacturer instructions (Illumina, San Diego, CA, USA). Sequencing was performed using V2 (150 bp × 2) chemistry. A single species (*Pseudomonas aeruginosa* DNA), a mock community (Zymo Microbial Community DNA Standard D6305), and a template-free negative control were included in the sequencing run.

The Qiime2 v2022.11 analysis package was used for sequence analysis [19]. The quality of the sequencing run was first examined using FastQC and MultiQC [20]. Cutadapt, with default settings, was used to remove sequences with high error rates [21]. Paired-end sequences were assembled and quality trimmed using vsearch –fastq_mergepairs [22, 23] with default settings and –fastq_truncqual set at 2, maxee set at 1, and minimum and maximum assemble lengths set at 250 and 255 (+ 2 and − 3 base pairs from the expected sequence length of 253 bp). Assembled sequences were subjected to an additional filtering step, utilizing the quality filter function in Qiime2. The resulting high-quality data was then processed using the deblur pipeline. Sequences were clustered into Amplicon Sequence Variant (ASV) groups and singleton sequences were removed. Taxonomy assignment was executed using the Qiime2 classify-hybrid-vsearch-sklearn function and the Average ReadyToWear trained Silva database version 138.1 [24, 25]. ASVs with an abundance of less than 0.01% were removed to reduce the potential for observing bleed-through ASVs, and ASVs identified as contaminating chloroplast or mitochondria were removed. A phylogenetic tree was created using the SEPP function available through Qiime2 [26]. The speciateIT tool was used to further annotate ASVs corresponding to major vaginal bacterial taxa to the species level. VALENCIA was then used to classify participants to their respective vaginal community state types (CST) as previously described elsewhere [27].

### qPCR analysis

qPCR was used to estimate total bacterial abundance. qPCR assays were TaqMan-based and performed on the QuantStudio 6 Flex Real-Time PCR System (Thermo Fischer Scientific). Primer and probe sequences are presented in Table S11 [28]. The total reaction volume for assays was 10 μL. Assays were performed at 95 °C for 10 min, 45 cycles at 95 °C for 15 s, and then at 60 °C for 1 min. Data analysis was performed with QuantStudio Real-Time PCR Software version 1.3 (Applied Biosystems) and the ThermoFisher Connect platform. Lab-grown pure cultures of *Prevotella bivia* were used for standard curves and to determine the LLOQ, defined as the lowest duplicates with reportable CT values. *P. bivia* was grown on TSA agar with 5% sheep’s blood (Hardy Diagnostics) anaerobically (80% N_2_; 10% CO_2_; 10 H_2_) at 37 °C for 46–48 h. Concentrations below the LLOQ were set to the LLOQ. All final values were normalized to the volume of the CVS collected originally and reported as ng of DNA/mL of CVS.

### Statistics

All statistical tests were performed in either R v4.3.2 or Graphpad Prism v10.2.0. Total bacterial load and soluble immune factors were compared between CSTs with one-way ANOVA with Tukey post-hoc tests. Associations between total bacterial load, estimated absolute abundance of individual bacterial taxa (calculated by multiplying the relative abundance of a taxon by total bacterial load), and soluble immune factors were measured with linear regression. Comparison of linear regression models was performed with the *anova* function in R. Total bacterial load was compared between BV-positive and BV-negative women (defined as either Nugent score ≥ 7 or CST-IV) with the Mann–Whitney *U* test. The association between all estimated bacterial absolute abundances and immune factors was performed with MaAsLin2 v 1.16.0. Specific parameters of each MaAsLin2 model were as follows: fixed_effects = individual soluble immune factors, analysis_method = “LM”, normalization = “NONE”, and transform = “NONE”. *p* values from the MaAsLin2 analysis were corrected for multiple comparisons with the false discovery rate. Total bacterial load was evaluated as a predictor of Nugent BV (Nugent score ≥ 7) and molecular BV (CST-IV) using logistic regression. Bacterial load, Nugent BV status, and CST were evaluated as predictors of soluble immune factors with linear regression. The performance of the models was compared using Vuong’s test (“performance” v0.11.0 package in R). Principal component analyses (PCA) were performed using the “FactoMineR” v2.11 package in R for complete data sets or the “missMDA” v1.19 package in R in the case of missing data. PCA plots were visualized with the “factoextra” v1.0.7 package in R. Partial around medoid (PAM) clustering was performed with the “cluster” v3.7–0 package in R. Optimal number of PAM clusters was determined using silhouette analysis with the “factoextra” v1.0.7 package in R.

## Results

### Participant characteristics and vaginal microbiota characterization

**Fig. 1:**
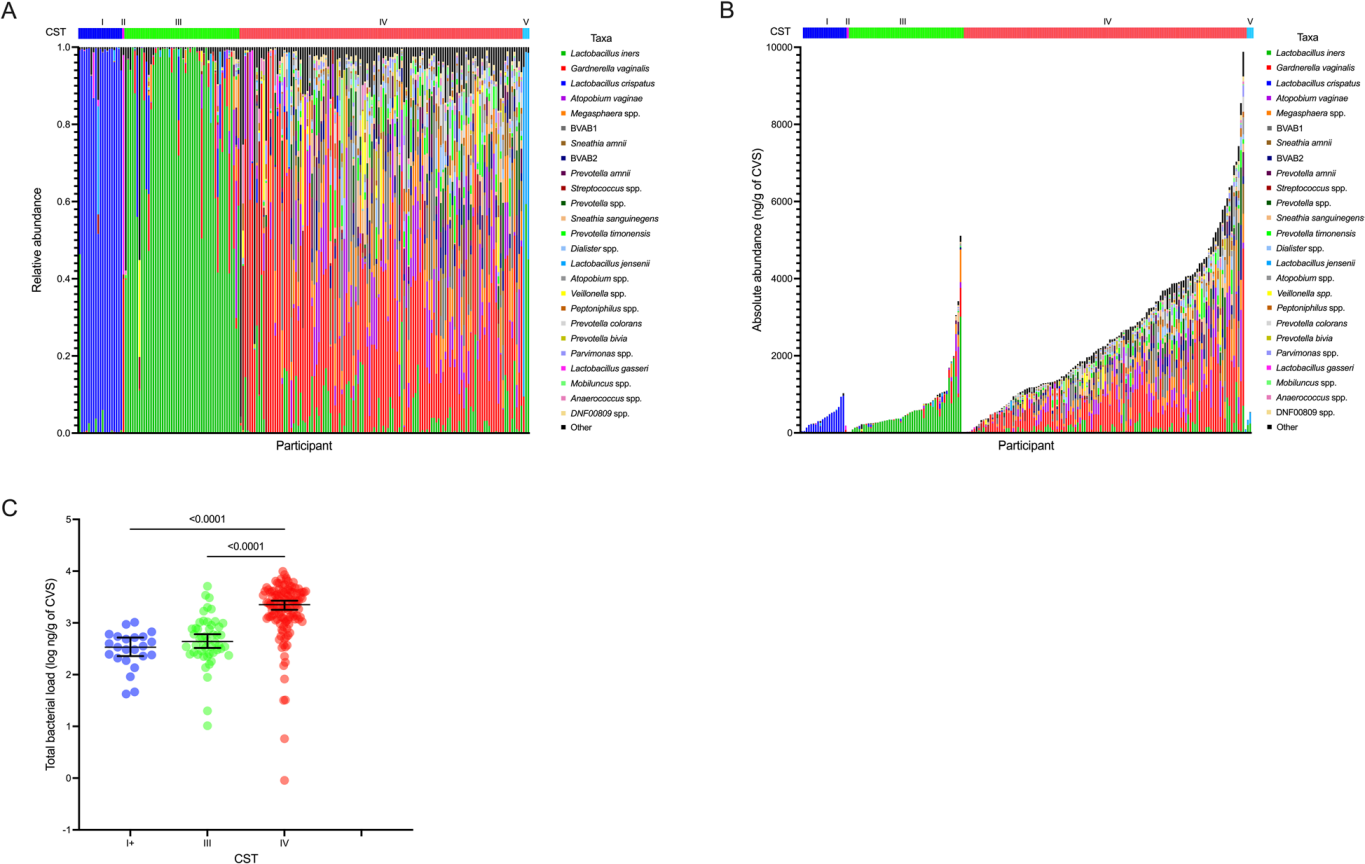
High variability of vaginal bacterial load across community state types. **A** Relative abundance of the top 25 most abundant organisms identified by 16S rRNA gene sequencing, sorted by CST. **B** Absolute abundance of the top 25 most abundant organisms identified by 16S rRNA gene sequencing, sorted by CST. **C** Comparison of total vaginal bacterial load between major CSTs. *p* values were obtained with Tukey post-hoc tests following one-way ANOVA

We characterized immune and microbial parameters in cervicovaginal secretions (CVS) from 196 non-pregnant, HIV-uninfected, and bacterial STI-free women. Participants were randomly selected from an ongoing cohort study of women who sell sex in Nairobi [29], with deliberate enrichment of participants with BV (90/196; 46%) or intermediate vaginal microbiota (43/196; 22%) by Nugent score. The median age of participants was 30 years (range 19–45 years) and 97/196 (49%) participants reported using hormonal contraception (Table 1). Participants with classical STIs were excluded, and approximately half of the participants were seropositive for HSV-2 (106/196; 54%). We characterized the bacterial composition of the vaginal microbiota with 16S rRNA gene sequencing and identified five vaginal community state types (CSTs) using the VALENCIA classification tool [27]. CST-I (*L. crispatus* predominance) was observed in 19/196 (10%) of women, CST-II (*Lactobacillus gasseri* predominance) in 1/196 (0.5%) of women, CST-III (*Lactobacillus iners* predominance) in 50/196 (26%) of women, CST-IV (diverse, BV-type) in 123/196 (63%) of women, and CST-V (*Lactobacillus jensenii* predominance) in 3/196 (2%) of women (Fig. 1A). Sociodemographic characteristics did not differ significantly between participants with these CSTs (Table S1). Given the small number of participants with CST-II or CST-V, these participants were pooled with CST-I for subsequent analyses, consistent with previous studies [4, 5]. Henceforth, we refer to this composite group as “CST-I + ”.

**Table 1:** Sociodemographic characteristics

Variable	Median or number (range or %)
Age	30 (19–45)
Contraceptive implant	34 (17%)
Injectable contraceptive	33 (17%)
IUD	5 (3%)
Oral contraceptive	25 (13%)
HSV-2 positive	106 (54%)
Syphilis positive	3 (2%)
Douching	94 (48%)
Days since the last vaginal sex	3 (0–7)
Number of clients in past seven days	3 (0–10)
Casual clients	169 (86%)
Regular clients	184 (94%)
Non-paying sex partner	128 (65%)

### Vaginal microbiota composition is strongly linked with total bacterial load

Quantitative profiles of the vaginal microbiota were generated by multiplying the bacterial relative abundances obtained from 16S rRNA gene sequencing by total bacterial load as quantified by qPCR, resulting in estimated absolute abundances of all bacterial taxa identified with 16S rRNA gene sequencing. Taxa with relative abundances less than 1% were assigned a value of zero to limit over-inflation of low abundance taxa and improve the accuracy of absolute abundance estimation, as previously validated by Tettamanti Boshier and colleagues [30]. There was considerable variation in total bacterial load within and between CSTs (Fig. 1B), with significantly higher total bacterial load among women with CST-IV compared to CST-I + and CST-III (both *p* < 0.0001). Although total bacterial load did not differ between CST-III and CST-I + , a subset of women within CST-III exhibited an intermediate total bacterial load between CST-I + and CST-IV (Fig. 1B, C). Given the wide variation in bacterial load that was apparent within CST-IV, we explored whether this variation was driven by compositional subgroups. VALENCIA identified four CST-IV subgroups in this cohort: CST-IV-A (moderate to high relative abundance of BV-associated bacterium 1 (BVAB1) and *Gardnerella vaginalis*) in 30/123 (24%) of women, CST-IV-B (moderate to high relative abundance of *G. vaginalis* and *Fannyhessea vaginae*) in 87/123 (71%) of women, CST-IV-C1 (*Streptococcus* spp. predominance) in 5/123 (4%) of women, and CST-IV-C4 (*Staphylococcus* spp. predominance) in 1/123 (1%) of women [27]. Given the low prevalence of CST-IV-C1 and CST-IV-C4, we combined these groups into “CST-IV-C” for subsequent statistical analyses. Total bacterial load was comparable among CST-IV-A and CST-IV-B, who represented the majority of women in CST-IV (117/123, 95%) within CST-IV. Bacterial load was lower in CST-IV-C, although there were relatively few participants in this subgroup (6/123, 5%; Fig. S1).

### Vaginal microbiota composition and bacterial load are associated with soluble immune factors

**Fig. 2:**
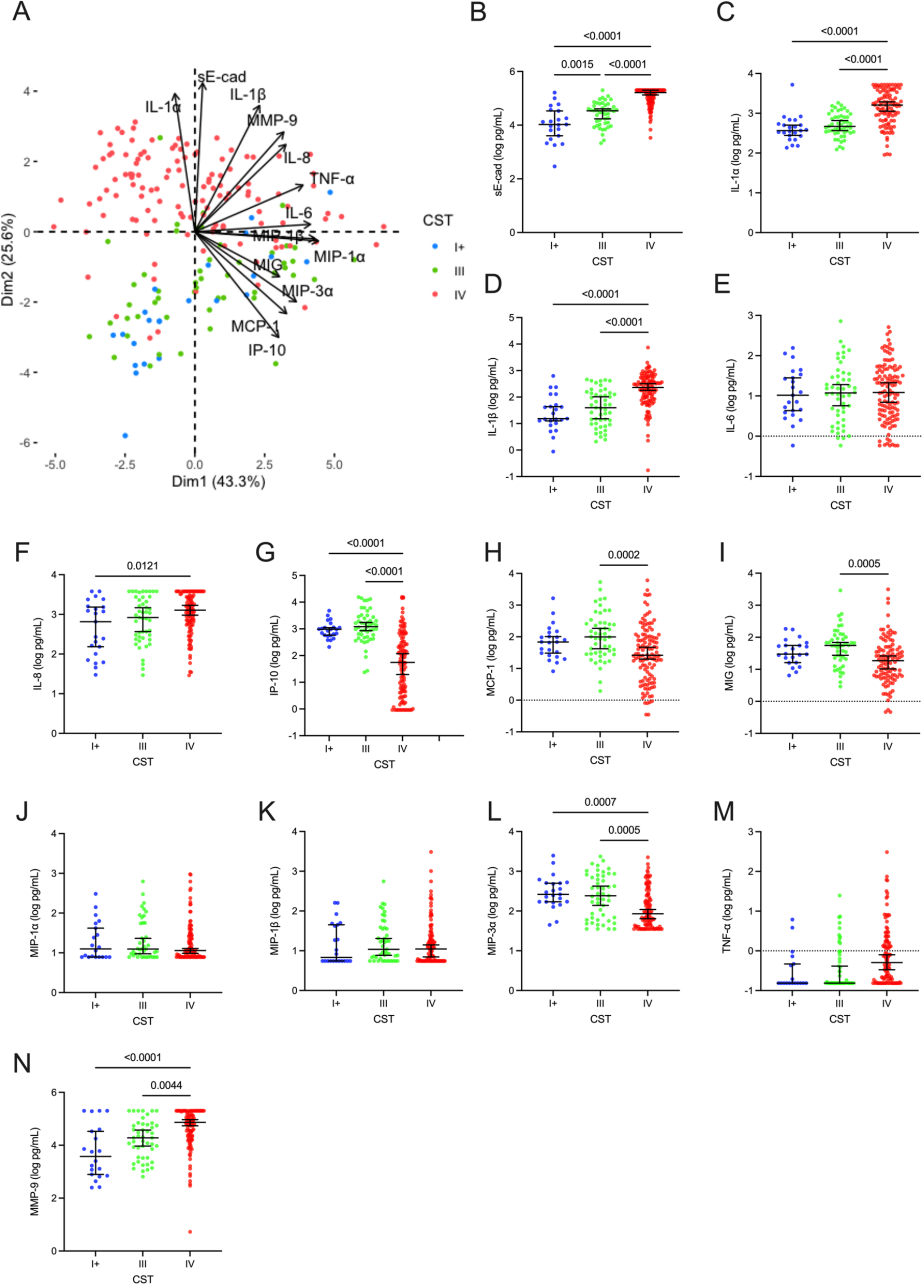
Vaginal soluble immune factors differ based on vaginal microbiota composition. Comparison of vaginal levels of **A** sE-cad, **B** IL-1α, **C** IL-1β, **D** IL-6, **E** IL-8, **F** IP-10, **G** MCP-1, **H** MIG, **I** MIP-1α, **J** MIP-1β, **K** MIP-3α, **L** TNF-α, and **M** MMP-9 between CST-I/II/V, CST-III, and CST-IV. *p* values obtained with Tukey post-hoc tests following one-way ANOVA

Levels of vaginal soluble immune factors were similar in CST-I + and CST-III except for levels of sE-cad, a biomarker of epithelial disruption, which were higher in CST-III (*p* = 0.0015). CST-IV was immunologically very different from CST-I + and CST-III, being characterized by higher levels of sE-cad, IL-1α, IL-1β, IL-8, and MMP-9 and lower levels of IP-10, MIP-3α, MIG, and MCP-1 compared (Fig. 2). Given the heterogeneity in total bacterial load between CST subgroups within CST-IV, we also compared levels of soluble immune factors between CST-IV subgroups. Consistent with our bacterial load comparisons, CST-IV-A and CST-IV-B had comparable immune profiles. CST-IV-C, which had relatively low bacterial load across CST-IV subgroups, also had elevated levels of vaginal chemokines, including IP-10, MCP-1, MIG, MIP-1α, MIP-1β, and MIP-3α, and elevated TNF-α (Fig. S1).

**Fig. 3:**
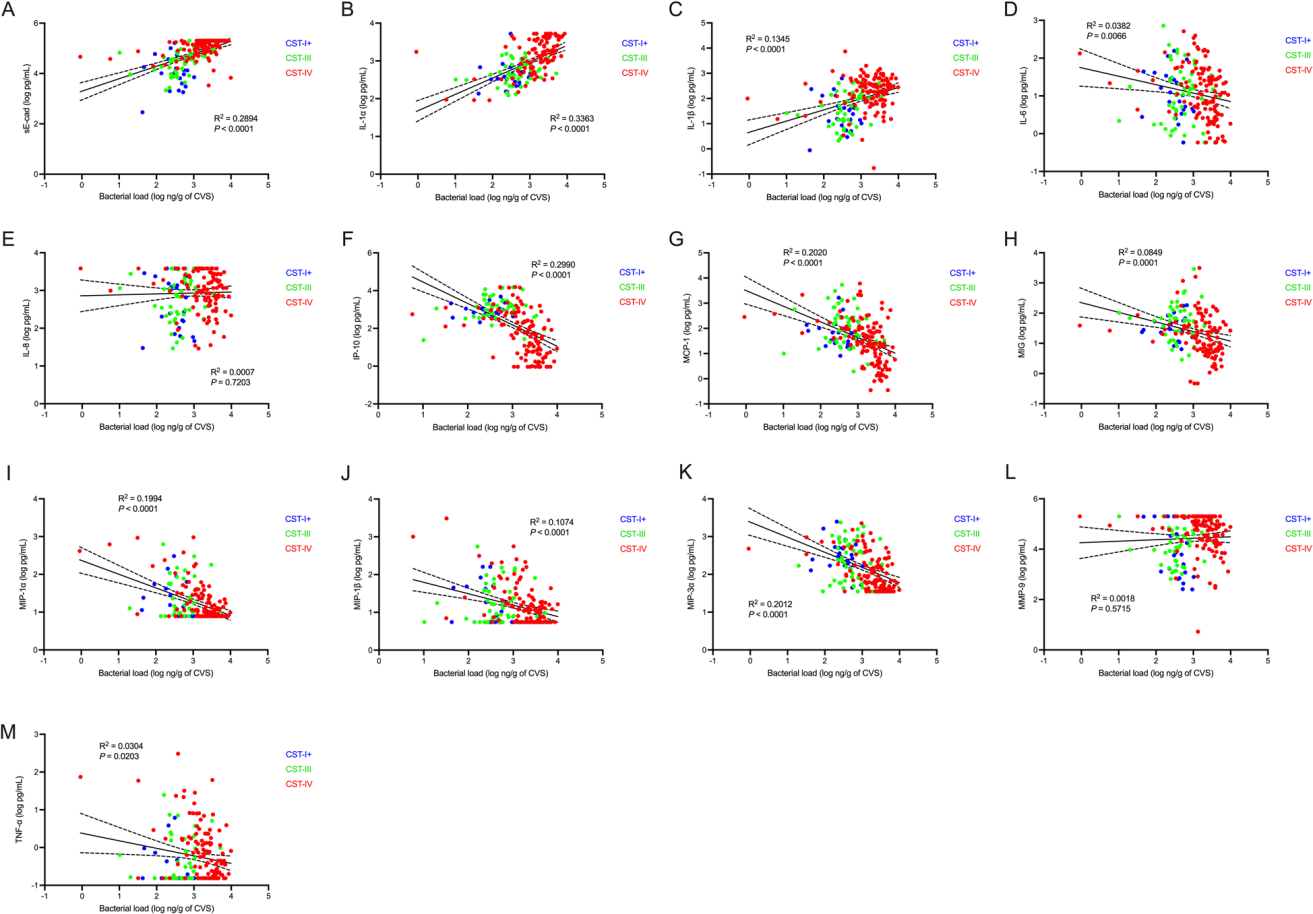
Vaginal bacterial load is associated with vaginal immune factors. Association between total bacterial load and **A** sE-cad, **B** IL-1α, **C** IL-1β, **D** IL-6, **E** IL-8, **F** IP-10, **G** MCP-1, **H** MIG, **I** MIP-1α, **J** MIP-1β, **K** MIP-3α, **L** TNF-α, and **M** MMP-9. Data points are colored by CST. *p* values and test statistics were obtained with linear regression

To explore how the association between vaginal microbiota composition and genital immunology might be affected by total bacterial load, we generated linear regression models evaluating the association between vaginal bacterial load and soluble immune factors. The immune associations of total bacterial load closely mirrored those seen with CST. Specifically, there were positive associations between absolute bacterial load and vaginal levels of sE-cad (*R*^2^ = 0.2894, *p* < 0.0001), IL-1α (*R*^2^ = 0.3363, *p* < 0.0001) and IL-1β (*R*^2 ^= 0.1345, *p* < 0.0001), while there were negative associations and with IL-6 (*R*^2 ^= 0.0382, *p* = 0.0066), IP-10 (*R*^2^ = 0.2990, *p* < 0.0001), MCP-1 (*R*^2 ^= 0.2020, *p* < 0.0001), MIG (*R*^2^ = 0.0849, *p* = 0.0001), MIP-1α (*R*^2 ^= 0.1994, *p* < 0.0001), MIP-1β (*R*^2 ^= 0.1074, *p* < 0.0001), MIP-3α (*R*^2^ = 0.2012, *p* < 0.0001), and TNF-α (*R*^2^ = 0.0304, *p* = 0.0203; Fig. 3).

### Total bacterial load modifies the relationship between vaginal microbiota composition and genital immunology

**Fig. 4:**
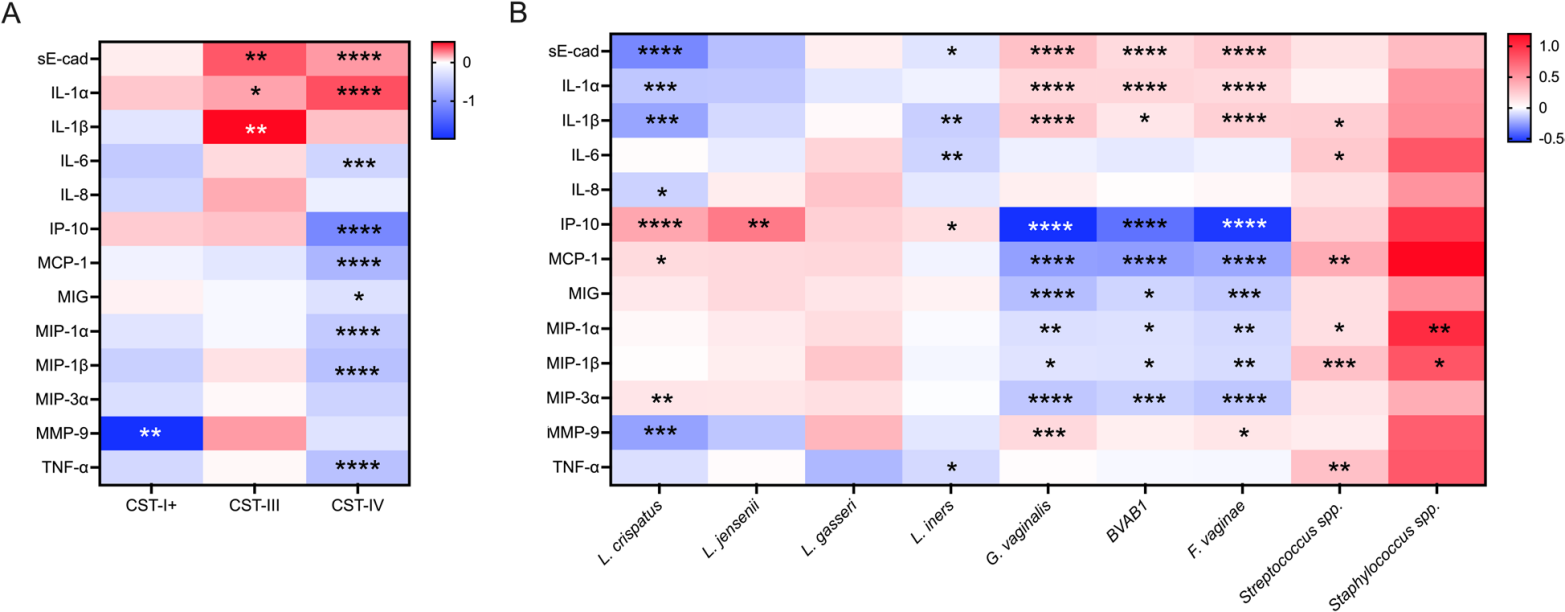
Relationship between vaginal bacterial load and genital immunology varies by vaginal microbiota composition. Heatmap displaying the association between **A** total bacterial load and vaginal immune factors, stratified by CST, and **B** estimated absolute abundance of key bacterial taxa and vaginal immune factors. Cells are shaded according to linear regression coefficients and annotated based on *p* values. *p < 0.05, **p < 0.01, ***p < 0.001, ****p < 0.0001

Given the association between vaginal microbiota composition (CSTs) and absolute bacterial load, we next examined whether the association between absolute bacterial load and genital immunology was dependent on vaginal microbiota composition. We evaluated the association between total bacterial load and immune factors independently for each vaginal CST. Among participants with CST-III, the absolute bacterial load was positively associated with sE-cad (*R*^2^ = 0.1441, *p* = 0.0093), IL-1α (*R*^2^ = 0.1018, *p* = 0.0239), and IL-1β (*R*^2^ = 0.1365, *p* = 0.0090). Likewise, among participants with CST-IV, total absolute bacterial load was positively associated with vaginal levels of sE-cad (*R*^2^ = 0.1319, *p* < 0.0001) and IL-1α (*R*^2^ = 0.2589, *p* < 0.0001). Negative associations between bacterial load and IL-6 (*R*^2^ = 0.1125, *p* = 0.0002), IP-10 (*R*^2 ^= 0.2303, *p* < 0.0001), MCP-1 (*R*^2 ^= 0.2111, *p* < 0.0001), MIG (*R*^2 ^= 0.0585, *p* = 0.0125), MIP-1α (*R*^2^ = 0.3914, *p* < 0.0001), MIP-1β (*R*^2 ^= 0.2986, *p* < 0.0001), MIP-3α (*R*^2^ = 0.2356, *p* < 0.0001), MMP-9 (*R*^2^ = 0.0412, *p* = 0.0281), and TNF-α (*R*^2^ = 0.2025, *p* < 0.0001) were also observed in CST-IV (Fig. 4). In contrast, among participants with CST-I + there was an inverse association between bacterial load and MMP-9 (*R*^2^ = 0.4096, *p* = 0.0024) and similar trends with IL-6 (*R*^2 ^= 0.0889, *p* = 0.1679), MIP-1β (*R*^2 ^= 0.1007, *p* = 0.1502), and MIP-3α (*R*^2 ^= 0.0778, *p* = 1974). Similar patterns were observed when evaluating the association between immune factors and the estimated absolute abundance of key bacterial taxa that are hallmarks of each CST. *L. crispatus* was negatively associated with proinflammatory cytokines and positively associated with the chemokines IP-10 and MCP-1, while the classic BV-associated bacteria *Gardnerella vaginalis*, BVAB1, and *F. vaginae* were positively associated with sE-cad and proinflammatory cytokines and negatively associated with chemokines like IP-10. Interestingly, *L. iners* absolute abundance was negatively associated with sE-cad and proinflammatory cytokines which is in contrast to the association between total bacterial load and immune factors within CST-III, suggesting that bacteria other than *L. iners* may be important determinants of genital immunology within CST-III. To explore this relationship further, we selected all participants within CST-III and employed MaAsLin2 to evaluate whether the estimated absolute abundance of any bacterial taxa was associated with sE-cad, IL-1α, or IL-1β. After correcting for multiple comparisons, the absolute abundance of BV-associated bacteria, including *G. vaginalis* and *F. vaginae*, but not *L. iners*, were positively associated with sE-cad and IL-1α (Fig. S2). Full results are presented in Table S2. Despite the relatively low prevalence of *Streptococcus* spp. and *Staphylococcus* spp., the characteristic taxa of CST-IV-C1 and CST-IV-C4, both taxa were positively associated with a variety of proinflammatory cytokines and chemokines (Fig. 4). Together, these results suggest that absolute bacterial load and vaginal microbiota composition are inextricably linked and that each serves as an important and complementary determinant of the female genital immune milieu.

### Absolute bacterial load alone independently predicts BV status and genital immunology

**Fig. 5:**
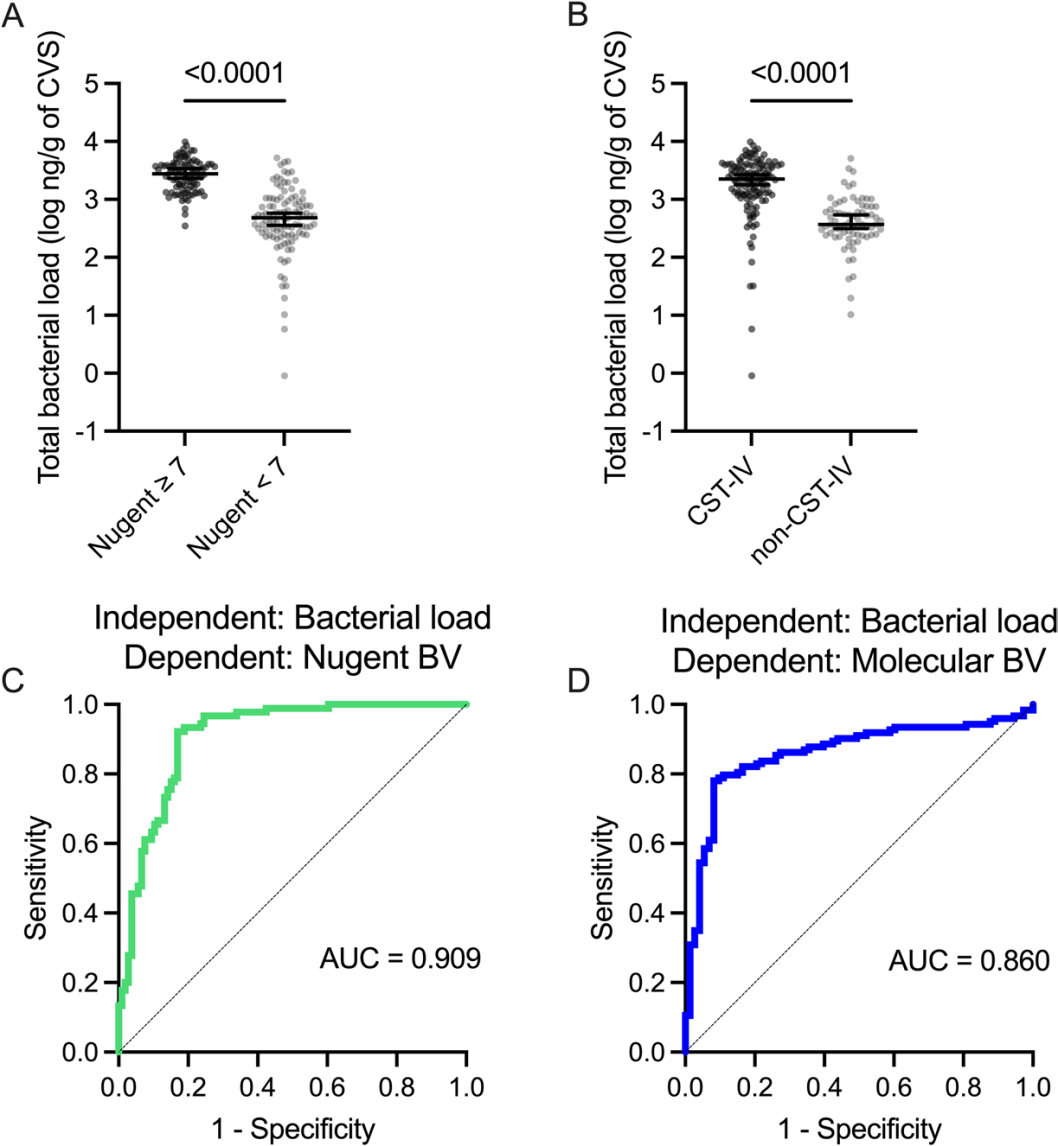
Vaginal bacterial load is a predictor of non-optimal vaginal microbiota states. Comparison of total vaginal bacterial load based on **A** Nugent BV and **B** molecular BV status. Receiver operator characteristic (ROC) curves for the prediction of **C** Nugent BV and **D** molecular BV with total bacterial load. *p* values were obtained with the Mann–Whitney *U* test or logistic regression

Although qPCR-based techniques have been implemented as diagnostic tools for BV, they require a priori selection of bacterial targets which may not be present in all clinical cases of BV. Non-specific quantification of absolute vaginal bacterial load might avoid this barrier while retaining the cost-effectiveness and sensitivity of qPCR. To this end, we evaluated whether total bacterial load alone could distinguish between important clinical phenotypes. We defined BV either based on a Nugent score ≥ 7 (Nugent BV) or classification as CST-IV according to 16S rRNA gene sequencing (molecular BV). Total bacterial load was higher among women with Nugent BV (*p* < 0.0001) and molecular BV (*p* < 0.0001) compared to the respectively defined BV-negative women. In logistic regression models, total bacterial load was strongly predictive of Nugent BV (*p* < 0.0001, AUC = 0.909) and molecular BV (*p* < 0.0001, AUC = 0.860; Fig. 5). Among participants who were misclassified by the model predicting Nugent BV, women with intermediate Nugent scores (i.e., 4–6) were enriched among the false positives and women with Nugent scores of 7–8 were enriched among the false negatives (Table S3). In the molecular BV prediction model, CST-III-A and CST-III-B were enriched among the false positives, and CST-IV-B, CST-IV-C1, and CST-IV-C4 were enriched among the false negatives (Table S4).

Next, we sought to benchmark total bacterial load as a predictor of the genital immune milieu compared to clinical standards (i.e., Nugent BV) and molecular characterization (i.e., vaginal CSTs). We generated linear regression models that incorporated total bacterial load, Nugent BV status, and vaginal CST, either alone or in combination with one another, to predict vaginal levels of sE-cad, IL-1α, and IP-10 since these immune factors were most strongly associated with vaginal microbiota composition and total bacterial load. Total bacterial load, Nugent BV, and CST were all independently predictive of each immune factor (adjusted *R*^2^ > 0.25, *p* < 0.0001 for each). Compared to Nugent BV and CST, total bacterial load was an equally strong predictor of IL-1α and IP-10 levels. The only difference in predictive ability was observed in models predicting sE-cad, where CST explained significantly more variance than total bacterial load (*p* = 0.006). For each immune factor, the models with the greatest explanatory power were those that included combinations of more than one vaginal microbiota descriptor as the predictors (Table S5), indicating that these methods of vaginal microbiota characterization are complementary determinants of the genital immune milieu. To evaluate the explanatory power of vaginal microbiota characterization techniques for the overall genital immune milieu, we employed partial around medoid (PAM) clustering based on levels of each immune factor. Since PAM clustering can only be performed for samples with complete data and many factors were unquantifiable in some samples, this analysis was limited to a subset of 111 participants with complete data available. Neither sociodemographic factors, Nugent BV prevalence, nor molecular BV (CST-IV) prevalence differed among participants with complete immune data compared to those without complete immune data (Table S6). Participants were clustered into two groups based on the optimal number of clusters determined with silhouette analysis, although visualization with principal component analysis (PCA) exhibited some overlap between clusters (Fig. S3). Immune clusters were not associated with sociodemographic characteristics (Table S7). Total bacterial load was a stronger predictor of these immune clusters than CST (*p* = 0.003) and tended to be a stronger predictor compared to Nugent BV status (*p* = 0.098; Table 2).

**Table 2:** Model comparison for the prediction of PAM immune clusters

Predictor	Tjur’s *R*^2^	*p* value	*p* value (compared to bacterial load model)
Bacterial load	0.478	< 0.0001	
Nugent BV	0.345	< 0.0001	0.098
CST	0.196	< 0.0001	0.003

Test statistics and associated *p* values were obtained with logistic regression. *p* values for model comparison were obtained with Vuong’s test

### Confirmation of bacterial load as a predictor of the genital immune milieu

**Fig. 6:**
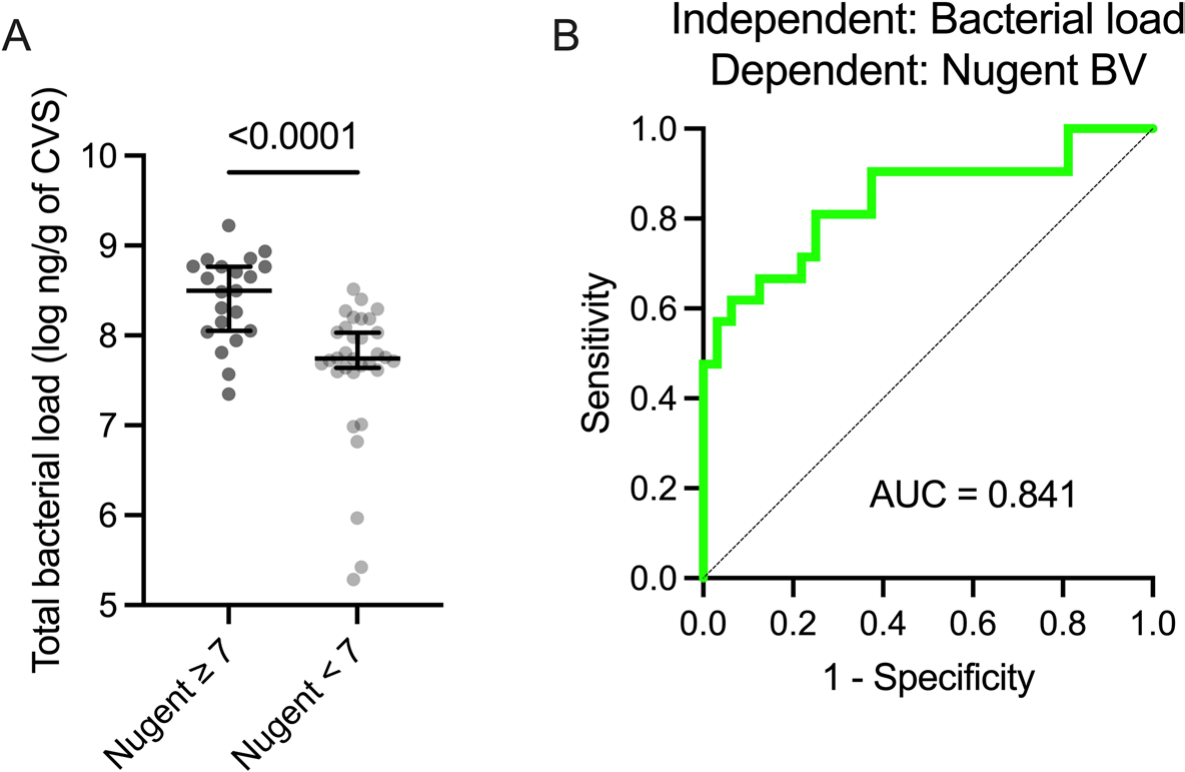
Bacterial load is a strong predictor of BV in an independent, Uganda-based cohort. Comparison of total bacterial load based on **A** Nugent BV and **B** ROC curves for the prediction of **B** Nugent BV with bacterial load. *p* values obtained with the Mann–Whitney *U* test or logistic regression

We then validated this analytic approach in an independent, previously published cohort of 53 non-pregnant, HIV-uninfected females from Uganda from whom total bacterial load and Nugent score data were available [31]. Sociodemographic factors for this cohort are presented in Table S8. In this Uganda-based cohort, absolute bacterial load was again increased in women with Nugent BV (*p* < 0.0001) and was a very strong predictor of Nugent BV (*p* < 0.0001, AUC = 0.841; Fig. 6). 16S rRNA gene sequencing was not performed on samples from the Uganda cohort, so we could not test whether vaginal bacterial load was predictive of molecular BV. Among participants who were misclassified in the model, all false positives had a Nugent score of 0 and most false negatives had a Nugent score of 8 (Table S9). We also compared total bacterial load, Nugent BV, and a combination of the two variables as predictors of the genital immune milieu. sE-cad was not measured in this cohort, so we generated linear regression models to predict vaginal levels of IL-1α and IP-10. Importantly, bacterial load was the only predictor that was significantly associated with IL-1α levels. Each model that predicted IP-10 levels exhibited comparable performance (Table S10). PAM clustering and silhouette analysis were then performed using immune factors that overlapped with those assayed in the Kenya cohort, resulting in three immune clusters (Fig. S4). Nugent BV and total bacterial load performed similarly as predictors of immune clusters #1 (*p* = 0.218) and #3 (*p* = 0.211), and total bacterial load tended to be a stronger predictor of immune cluster #2 (*p* = 0.073; Table 3).

**Table 3:** Model comparison for the prediction of PAM immune clusters in an independent, Uganda-based cohort

Dependent variable	Predictor	Estimate	*p* value	*p* value (comparison of models)
Immune cluster #1	Bacterial load	− 0.614	0.128	0.218
	Nugent BV	− 1.540	0.020	
Immune cluster #2	Bacterial load	3.904	0.0006	0.073
	Nugent BV	2.271	0.001	
**Immune cluter #3**	Bacterial load	-0.645	0.106	0.211
	Nugent BV	− 0.473	0.483	

Test statistics and associated *p* values were obtained with logistic regression. *p* values for model comparison obtained with Vuong’s test.

## Discussion

The composition of the vaginal microbiota is closely linked to adverse sexual and reproductive health outcomes such as HIV acquisition and preterm birth, likely through the modulation of genital mucosal inflammation [3, 4]. However, many studies in the field have relied primarily on relative, rather than quantitative, methods to characterize the vaginal microbiota. Our results demonstrate that vaginal bacterial load and microbiota composition are both determinants of the genital immune milieu, yielding complementary associations with vaginal soluble immune factors. We also show that the total bacterial load as a single variable performs comparably to multivariable vaginal microbiota characterization as a predictor of the genital immune milieu.

The relationship between the bacterial composition of the vaginal microbiota and genital immunology has been explored extensively, but often relies on relative compositional data. To expand upon this paradigm, we generated quantitative microbiota profiles, similar to previous studies of the gut microbiota [11, 12]. Vaginal bacterial load differed dramatically based on overall vaginal microbiota composition, and the absolute bacterial load was also associated with a variety of vaginal immune factors in a composition-dependent manner. These findings build upon a growing body of literature that highlights the importance of quantitative methods in the elucidation of host-microbe interactions in vivo in a variety of mucosal sites [11, 12, 32]. However, the majority of these studies have focused on the gastrointestinal tract, while our work highlights the importance of exploring this relationship in greater detail in the female genital tract and suggests that quantitative methods may represent a vital complement to compositional methods and expand upon our understanding of host-microbe interactions. An example of how quantitative profiling of the vaginal microbiota can complement compositional methods is the case of *L. iners* predominance. Many groups agree that *L. iners* predominance is not favorable compared to other *Lactobacillus* species like *L. crispatus*, but the genital immune profile of women with *L. iners* predominance is often (including in the current study) similar to that of women with non-*iners Lactobacillus* predominance [5]. By incorporating quantitative methods, we show that bacterial load is positively associated with proinflammatory cytokines and a marker of epithelial disruption among women with *L. iners* predominance, although most likely driven by the absolute abundance of co-colonizing BV-associated bacteria, not among women with non-*iners Lactobacillus* predominance. The incorporation of quantitative methods also provided insight into the potential mechanisms underpinning heterogenous genital cytokine profiles within CST-IV. While a BV-type vaginal microbiome, such as CST-IV, is commonly associated with elevated genital proinflammatory cytokine levels, there remains considerable unexplained heterogeneity in proinflammatory cytokine levels within CST-IV, which may translate into heterogeneity in HIV risk among women in CST-IV [14]. Several studies have explored potential reasons for this heterogeneity, including variability in proinflammatory cytokine induction by different BV-associated microbes [5] and differential mucosal antibody responses to BV-associated bacteria [33]. We show that total bacterial load within CST-IV may explain a considerable amount of this variability. To our knowledge, this is the first study to demonstrate the importance of bacterial absolute abundances as potential drivers of heterogeneity in genital cytokine profiles within the same vaginal microbiome community state.

BV can be difficult to diagnose in clinical and research settings, due in part to its inherent heterogeneity and lack of a single etiologic agent [34]. qPCR-based diagnostic strategies have emerged as possible improvements upon current clinical tools, such as Amsel or Nugent scoring [35]. However, these qPCR-based strategies require a priori selection of microbial targets. This is problematic because of the high inter-individual heterogeneity in the bacterial composition of BV, which is difficult to predict without prior knowledge of the microbial community. In addition, HIV risk among BV-negative women is heterogenous, with predominance by *L. iners* believed to confer less protection against HIV acquisition than non-*iners Lactobacillus* species. Given the association between BV and vaginal bacterial load in our analysis and previous studies [14], we hypothesized that quantifying total bacterial load, rather than the absolute abundance of specific bacterial taxa, would retain the sensitivity of qPCR-based approaches without the need for a priori selection of bacterial targets. We show that total bacterial load is a strong predictor of the clinical diagnostic standard for BV (Nugent BV) and molecular BV as defined by 16S rRNA gene sequencing. We also show that bacterial load alone performs equally well, and sometimes better, as a predictor of the genital immune milieu compared to Nugent BV and extensive molecular characterization of the vaginal microbiota. Therefore, vaginal bacterial load quantification with qPCR may represent a relatively rapid, inexpensive, and widely implementable method of identifying non-optimal vaginal microenvironments. Given the increased focus on vaginal microbiota-targeting therapeutics, bacterial load-based assessments may provide complementary analyses to relative abundance-only methods such as amplicon and shotgun metagenomic sequencing in observational and interventional studies. For example, interventional trials may benefit from incorporating total vaginal bacterial load as a screening tool to identify women who might benefit from such interventions or for assessing the mediation of therapeutic outcomes.

This study has several important limitations. First, our studies were performed in a cohort study recruiting female sex workers and were enriched for participants with BV. However, we reproduce many of our findings in a confirmatory cohort of women based in Uganda, underscoring their likely generalizability in African women. Second, the cross-sectional nature of our analysis prevented us from elucidating causal mechanisms, including the relative importance of microbiota composition and bacterial load in driving genital immunology. We encourage future studies to elucidate potential causal relationships in greater detail by employing in vitro and longitudinal methods. We also recognize several potential limitations of a diagnostic approach that only employs total bacterial load quantification. For example, misclassification of participants with high bacterial load and CST-I + or CST-III or low bacterial load and CST-IV would be difficult to avoid with such an approach. This relationship is reflected by the enrichment of CST-III in the false positives and CST-IV-C in the false negatives of our molecular BV prediction model since a subset of women in CST-III exhibited higher bacterial load whereas CST-IV-C was characterized by lower bacterial load. Importantly, we did not observe any women who would fit a “high” bacterial load phenotype among women with CST-I, although this should be explored in greater detail in diverse cohorts since *L. crispatus* is underrepresented among African, Caribbean, and other Black (ACB) women [36]. In addition, the selection of bacterial load cutoffs for diagnosing BV may require a more nuanced approach across the spectrum of BV diagnostic scores or vaginal CSTs. However, our sample sizes limited our ability to extensively stratify the present cohorts and we encourage future studies to explore this relationship in greater detail. Our analysis of the genital immune milieu was also limited to a panel of vaginal soluble immune factors. Given the diversity of genital mucosal immune factors that have important implications for sexual and reproductive health, we encourage future studies to explore the relationship between vaginal microbiome composition, bacterial load, and the genital immune milieu in greater detail by assessing more genital immune factors (e.g., immune cell subsets).

## Conclusions

This study expands upon well-established connections between vaginal microbiota composition and genital immunology by outlining a novel role for bacterial load as a determinant of this relationship. Specifically, we show that bacterial load alone is strongly associated with genital immunology and that this relationship differs based on total vaginal microbiota composition. Importantly, our results do not diminish the importance of individual microbes and microbiota composition as determinants of genital immunology and reproductive health. Rather, we believe that vaginal microbiota composition and bacterial load are complementary determinants of the genital immune milieu and that this observation may introduce new diagnostic avenues for the use of bacterial load quantification as an additional approach to clinical and sequencing-based techniques for the identification of vaginal microbial dysbiosis that are not limited to the detection of BV.

## Supplementary Information

Supplementary Material 1: Supplementary methods. Figure S1: Variation in total bacterial load and vaginal soluble immune factors across CST-IV subgroups. Figure S2: BV-associated bacteria drive the association between total bacterial load and immune factors within CST-III. Figure S3: Genital immune milieu cluster tightly with vaginal microbiota composition. Figure S4: Genital immune milieu is closely tied to vaginal microbiota composition in an independent, Uganda-based confirmatory cohort. Table S1: Association between vaginal CST and sociodemographic variables. Table S2: The absolute abundance of BV-associated bacteria, including *G. vaginalis* and *F. vaginae*, but not *L. iners*, were positively associated with sE-cad and IL-1α. Table S3: Nugent scores of women misclassified by the logistic regression model predicting Nugent BV with bacterial load in the SWOP cohort. Table S4: CST subgroups of women misclassified by the logistic regression model predicting molecular BV with bacterial load in the SWOP cohort. Table S5: Comparison of linear regression models predicting soluble immune factors with different vaginal microbiota characterization metrics. Table S6: Comparison of sociodemographic characteristics based on availability of complete immune data in the SWOP cohort. Table S7: Association between PAM immune cluster and sociodemographic variables. Table S8: Sociodemographic factors for Uganda-based confirmatory cohort. N = 61. Table S9: Nugent scores of women misclassified by the logistic regression model predicting Nugent BV with bacterial load in the Uganda-based confirmatory cohort. Table S10: Comparison of linear regression models predicting soluble immune factors with different vaginal microbiota characterization metrics in the confirmatory Uganda-based confirmatory cohort. Table S11: Primer and probe sequences for qPCR assays quantifying total bacterial load.

## Data Availability

Raw sequencing files have now been submitted to the NCBI SRA under the BioProject ID PRJNA1116725.

## References

[1] Ma B, Forney LJ, Ravel J. The vaginal microbiome: rethinking health and diseases. Annu Rev Microbiol. 2012;66:371–89. 22746335 10.1146/annurev-micro-092611-150157 PMC3780402

[2] Low N, Chersich MF, Schmidlin K, Egger M, Francis SC, van de Wijgert JHHM, et al. Intravaginal practices, bacterial vaginosis, and HIV infection in women: Individual participant data meta-analysis. PLoS Med. 2011;8:e1000416. 10.1371/journal.pmed.1000416 PMC3039685 21358808

[3] Fettweis JM, Serrano MG, Brooks JP, Edwards DJ, Girerd PH, Parikh HI, et al. The vaginal microbiome and preterm birth. Nat Med. 2019;25:1012–21. 31142849 10.1038/s41591-019-0450-2 PMC6750801

[4] Gosmann C, Anahtar MN, Handley SA, Farcasanu M, Abu-Ali G, Bowman BA, et al. Lactobacillus-deficient cervicovaginal bacterial communities are associated with increased HIV acquisition in young South African women. Immunity. 2017;46:29–37. 28087240 10.1016/j.immuni.2016.12.013 PMC5270628

[5] Anahtar MN, Byrne EH, Doherty KE, Bowman BA, Yamamoto HS, Soumillon M, et al. Cervicovaginal bacteria are a major modulator of host inflammatory responses in the female genital tract. Immunity. 2015;42:965–76. 25992865 10.1016/j.immuni.2015.04.019 PMC4461369

[6] Doerflinger SY, Throop AL, Herbst-Kralovetz MM. Bacteria in the vaginal microbiome alter the innate immune response and barrier properties of the human vaginal epithelia in a species-specific manner. J Infect Dis. 2014;209:1989–99. 24403560 10.1093/infdis/jiu004

[7] Amsel R, Totten PA, Spiegel CA, Chen KCS, Eschenbach D, Holmes KK. Nonspecific vaginitis. Diagnostic criteria and microbial and epidemiologic associations. Am J Med. 1983;74:14–22. 6600371 10.1016/0002-9343(83)91112-9

[8] Nugent RP, Krohn MA, Hillier SL. Reliability of diagnosing bacterial vaginosis is improved by a standardized method of gram stain interpretation. J Clin Microbiol. 1991;29:297–301. 1706728 10.1128/jcm.29.2.297-301.1991 PMC269757

[9] Gloor GB, Macklaim JM, Pawlowsky-Glahn V, Egozcue JJ. Microbiome datasets are compositional: and this is not optional. Front Microbiol. 2017;8:2224. 10.3389/fmicb.2017.02224 PMC5695134 29187837

[10] Armstrong E, Hemmerling A, Miller S, Burke KE, Newmann SJ, Morris SR, et al. Metronidazole treatment rapidly reduces genital inflammation through effects on bacterial vaginosis- associated bacteria rather than lactobacilli. J Clin Invest. 2022;132:e152930. 10.1172/JCI152930 PMC8920324 35113809

[11] Vandeputte D, Kathagen G, D’Hoe K, Vieira-Silva S, Valles-Colomer M, Sabino J, et al. Quantitative microbiome profiling links gut community variation to microbial load. Nature. 2017;551:507–11. 10.1038/nature24460 29143816

[12] Vieira-Silva S, Sabino J, Valles-Colomer M, Falony G, Kathagen G, Caenepeel C, et al. Quantitative microbiome profiling disentangles inflammation- and bile duct obstruction-associated microbiota alterations across PSC/IBD diagnoses. Nat Microbiol. 2019;4:1826–31. 10.1038/s41564-019-0483-9 31209308

[13] Beattie TS, Pollock J, Kabuti R, Abramsky T, Kung’u M, Babu H, et al. Are violence, harmful alcohol/substance use and poor mental health associated with increased genital inflammation?: a longitudinal cohort study with HIV-negative female sex workers in Nairobi, Kenya. PLOS Glob Publ Health. 2024;4:1–18. 10.1371/journal.pgph.0003592 PMC11349110 39190654

[14] Shannon B, Gajer P, Yi TJ, Ma B, Humphrys MS, Thomas-Pavanel J, et al. Distinct effects of the cervicovaginal microbiota and herpes simplex type 2 infection on female genital tract immunology. J Infect Dis. 2017;215:1366–75. 28201724 10.1093/infdis/jix088 PMC5451606

[15] Lynn WA, Lightman S. Syphilis and HIV: a dangerous combination. Lancet Infect Dis. 2004;4:456–66. 10.1016/S1473-3099(04)01061-8 15219556

[16] Al-Sadi R, Engers J, Haque M, King S, Al-Omari D, Ma TY. Matrix Metalloproteinase-9 (MMP-9) induced disruption of intestinal epithelial tight junction barrier is mediated by NF-κB activation. PLoS ONE. 2021;16:e0249544. 10.1371/journal.pone.0249544 PMC8026081 33826658

[17] Liu R, Armstrong E, Constable S, Buchanan LB, Mohammadi A, Galiwango RM, et al. Soluble E-cadherin: A marker of genital epithelial disruption. Am J Reprod Immunol. 2023;89:e13674. 10.1111/aji.13674 36593681

[18] Caporaso JG, Lauber CL, Walters WA, Berg-Lyons D, Huntley J, Fierer N, et al. Ultra-high-throughput microbial community analysis on the Illumina HiSeq and MiSeq platforms. ISME J. 2012;6:1621–4. 10.1038/ismej.2012.8 PMC3400413 22402401

[19] Bolyen E, Rideout JR, Dillon MR, Bokulich NA, Abnet CC, Al-Ghalith GA, et al. Reproducible, interactive, scalable and extensible microbiome data science using QIIME 2. Nat Biotechnol. 2019;37:852–7. 10.1038/s41587-019-0209-9 PMC7015180 31341288

[20] Ewels P, Magnusson M, Lundin S, Käller M. MultiQC: Summarize analysis results for multiple tools and samples in a single report. Bioinformatics. 2016;32:3047–8. 10.1093/bioinformatics/btw354 PMC5039924 27312411

[21] Martin M. Cutadapt removes adapter sequences from high-throughput sequencing reads. EMBnet.journal. 2011;17:10.

[22] Rognes T, Flouri T, Nichols B, Quince C, Mahé F. VSEARCH: a versatile open source tool for metagenomics. PeerJ. 2016;4:e2584. 10.7717/peerj.2584 PMC5075697 27781170

[23] Edgar RC. Search and clustering orders of magnitude faster than BLAST. Bioinformatics. 2010;26:2460–1. 10.1093/bioinformatics/btq461 20709691

[24] Bokulich NA, Kaehler BD, Rideout JR, Dillon M, Bolyen E, Knight R, et al. Optimizing taxonomic classification of marker-gene amplicon sequences with QIIME 2’s q2-feature-classifier plugin. Microbiome. 2018;6:90. 10.1186/s40168-018-0470-z PMC5956843 29773078

[25] Kaehler BD, Bokulich NA, McDonald D, Knight R, Caporaso JG, Huttley GA. Species abundance information improves sequence taxonomy classification accuracy. Nat Commun. 2019;10:4643. 10.1038/s41467-019-12669-6 PMC6789115 31604942

[26] Janssen S, McDonald D, Gonzalez A, Navas-Molina JA, Jiang L, Xu ZZ, et al. Phylogenetic placement of exact amplicon sequences improves associations with clinical information. mSystems. 2018;3:e00021–18. 10.1128/mSystems.00021-18 PMC5904434 29719869

[27] France MT, Ma B, Gajer P, Brown S, Humphrys MS, Holm JB, et al. VALENCIA: a nearest centroid classification method for vaginal microbial communities based on composition. Microbiome. 2020;8:166. 10.1186/s40168-020-00934-6 PMC7684964 33228810

[28] Nadkarni MA, Martin FE, Jacques NA, Hunter N. Determination of bacterial load by real-time PCR using a broad-range (universal) probe and primers set. Microbiology (Reading). 2002;148:257–66. 11782518 10.1099/00221287-148-1-257

[29] Beksinska A, Jama Z, Kabuti R, Kungu M, Babu H, Nyariki E, et al. Prevalence and correlates of common mental health problems and recent suicidal thoughts and behaviours among female sex workers in Nairobi, Kenya. BMC Psychiatry. 2021;21:503. 10.1186/s12888-021-03515-5 PMC8518166 34649544

[30] TettamantiBoshier FA, Srinivasan S, Lopez A, Hoffman NG, Proll S, Fredricks DN, et al. Complementing 16S rRNA gene amplicon sequencing with total bacterial load to infer absolute species concentrations in the vaginal microbiome. mSystems. 2020;5:1–14. 10.1128/mSystems.00777-19 PMC7141891 32265316

[31] Yegorov S, Joag V, Galiwango RM, Good S V., Mpendo J, Tannich E, et al. Schistosoma mansoni treatment reduces HIV entry into cervical CD4+ T cells and induces IFN-I pathways. Nat Commun. 2019;10:2296. 10.1038/s41467-019-09900-9 PMC6534541 31127086

[32] Mayer BT, Srinivasan S, Fiedler TL, Marrazzo JM, Fredricks DN, Schiffer JT. Rapid and profound shifts in the vaginal microbiota following antibiotic treatment for bacterial vaginosis. J Infect Dis. 2015;212:793–802. 25676470 10.1093/infdis/jiv079 PMC4539900

[33] Liu R, Pollock J, Huibner S, Udayakumar S, Irungu E, Ngurukiri P, et al. Microbe-binding antibodies in the female genital tract: associations with the vaginal microbiome and genital immunology. J Immunol. 2024;213:1516–27. 39345194 10.4049/jimmunol.2400233

[34] McKinnon LR, Achilles SL, Bradshaw CS, Burgener A, Crucitti T, Fredricks DN, et al. The evolving facets of bacterial vaginosis: implications for HIV transmission. AIDS Res Hum Retroviruses. 2019;35:219–28. 30638028 10.1089/aid.2018.0304 PMC6434601

[35] Redelinghuys MJ, Geldenhuys J, Jung H, Kock MM. Bacterial vaginosis: current diagnostic avenues and future opportunities. Front Cell Infect Microbiol. 2020;10:354. 10.3389/fcimb.2020.00354 PMC7431474 32850469

[36] Ravel J, Gajer P, Abdo Z, Schneider GM, Koenig SSK, McCulle SL, et al. Vaginal microbiome of reproductive-age women. Proc Natl Acad Sci. 2011;108:4680–7. 20534435 10.1073/pnas.1002611107 PMC3063603

