# Supplementary material for: Quantitative profiling of the vaginal microbiota improves resolution of the microbiota-immune axis
Source: Microbiome. 2025 Feb 4;13:39. doi: 10.1186/s40168-025-02039-4 (PMC11792376; doi:10.1186/s40168-025-02039-4)
Supplement: Supplementary file 2 — Supplementary Material 1: Supplementary methods. Figure S1: Variation in total bacterial load and vaginal soluble immune factors across CST-IV subgroups. Figure S2: BV-associated bacteria drive the association between total bacterial load and immune factors within CST-III. Figure S3: Genital immune milieu cluster tightly with vaginal microbiota composition. Figure S4: Genital immune milieu is closely tied to vaginal microbiota composition in an independent, Uganda-based confirmatory cohort. Table S1: Association between vaginal CST and sociodemographic variables. Table S2: The absolute abundance of BV-associated bacteria, including G. vaginalis and F. vaginae, but not L. iners, were positively associated with sE-cad and IL-1α. Table S3: Nugent scores of women misclassified by the logistic regression model predicting Nugent BV with bacterial load in the SWOP cohort. Table S4: CST subgroups of women misclassified by the logistic regression model predicting molecular BV with bacterial load in the SWOP cohort. Table S5: Comparison of linear regression models predicting soluble immune factors with different vaginal microbiota characterization metrics. Table S6: Comparison of sociodemographic characteristics based on availability of complete immune data in the SWOP cohort. Table S7: Association between PAM immune cluster and sociodemographic variables. Table S8: Sociodemographic factors for Uganda-based confirmatory cohort. N = 61. Table S9: Nugent scores of women misclassified by the logistic regression model predicting Nugent BV with bacterial load in the Uganda-based confirmatory cohort. Table S10: Comparison of linear regression models predicting soluble immune factors with different vaginal microbiota characterization metrics in the confirmatory Uganda-based confirmatory cohort. Table S11: Primer and probe sequences for qPCR assays quantifying total bacterial load. [file 40168_2025_2039_MOESM1_ESM.zip › 250114_QMP_supplemental.docx]

**Title: Quantitative profiling of the vaginal microbiota improves resolution of the microbiota-immune axis**

**SUPPLEMENTAL MATERIALS**

**MATERIALS AND METHODS**

**Study design – Uganda-based cohort:** Women aged 18-45 years from Entebbe, Uganda, were recruited as previously described[1]. Briefly, a physician-selected subset of women presenting to the Outpatient Department at Entebbe General Hospital with fever (axillary temperature ≥37.5 °C) or self-report of fever in the previous 24 hours, and a positive thick smear for malaria were enrolled. Exclusion criteria included pregnancy, HIV infection, malaria infection, genital ulceration, active menstruation, or a positive test for classical STIs (*Neisseria gonorrhoeae*, *Chlamydia trachomatis*, *Treponema pallidum* (syphilis), or *Trichomonas vaginalis*). Details on sample collection has been previously described[2]. Briefly, cervicovaginal secretions were collected with a SoftCup and frozen at -80°C immediately after collection. Cervicovaginal secretions were used for soluble immune factor and bacterial load quantification. A vaginal swab was then collected for Gram staining and Nugent scoring.

**Soluble immune factor quantification – Uganda-based cohort:** Soluble immune factors were quantified with multiplex immunoassay on the Meso Scale Discovery platform as previously described[2]. For the present analysis, only analytes that overlapped with those measured in the Kenya-based cohort were included (i.e., IL-1α, ΙL-1β, IL-6, IL-8, IP-10, MIG, MIP-1α, MIP-1β, MIP-3α, and TNF-α.

**DNA extraction and total bacterial load quantification – Uganda-based cohort:**

DNA was isolated from 250uL of the CVS pellet using the DNEasy PowerSoil Pro Kit (Qiagen), according to the manufacturer’s instructions. Total bacterial load of cervicovaginal secretions was quantified with qPCR on the miSeq platform. qPCR assays were TaqMan-based and performed on the QuantStudio 6 Flex Real-Time PCR System (Thermo Fischer Scientific). Primer and probe sequences are presented in Table S11. The total reaction volume for assays was 10 μL. Assays were performed at 95°C for 10 minutes, 45 cycles at 95°C for 15 seconds, and then at 60°C for 1 minute. Data analysis was performed with QuantStudio Real-Time PCR Software version 1.3 (Applied Biosystems) and the ThermoFisher Connect platform. Lab-grown pure cultures of *Pseudomonas aeruginosa* were used for standard curves and to determine the LLOQ, defined as the lowest duplicates with reportable CT values. *P. aeruginosa* was grown on nutrient agar aerobically at 37°C for 24hrs. Concentrations below the LLOQ were set to the LLOQ. All final values were normalized to the volume of the CVS collected originally and reported as ng of DNA /mL of CVS.

**REFERENCES**

1. Yegorov S, Galiwango RM, Ssemaganda A, Muwanga M, Wesonga I, Miiro G, et al. Low prevalence of laboratory-confirmed malaria in clinically diagnosed adult women from the Wakiso district of Uganda. Malar J. 2016;15.
2. Yegorov S, Joag V, Galiwango RM, Good SV, Mpendo J, Tannich E, et al. Schistosoma mansoni treatment reduces HIV entry into cervical CD4+ T cells and induces IFN-I pathways. Nat Commun. 2019;10.

**FIGURES AND TABLES**

**
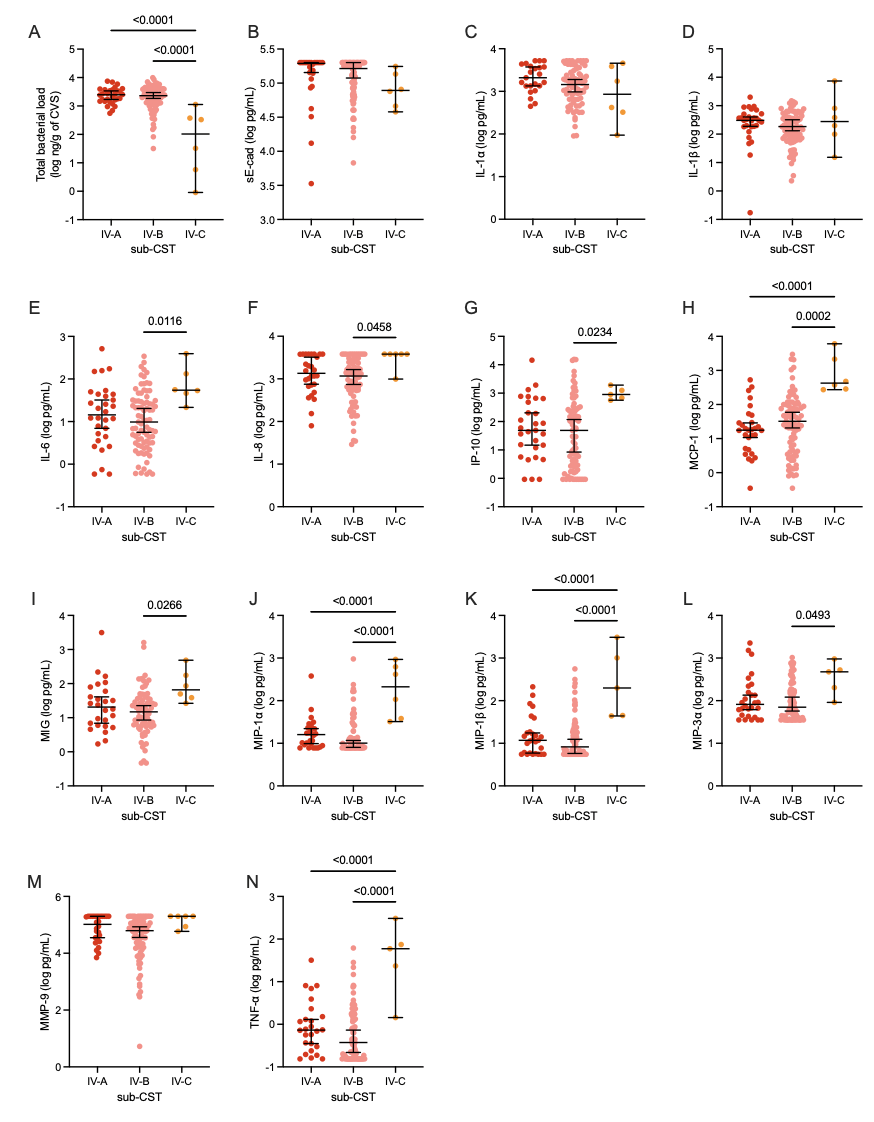
**

**Supplemental figure S1: Variation in total bacterial load and vaginal soluble immune factors across CST-IV subgroups.** Comparison of A) total vaginal bacterial load, B) sE-cad, C) IL-1α, D) IL-1β, E) IL-6, F) IL-8, G) IP-10, H) MCP-1, I) MIG, J) MIP-1α, K) MIP-1β, L) MIP-3α, M) MMP-9, and N) TNF-α between CST-IV-A, CST-IV-B, and CST-IV-C. *P* values generated with Tukey post-hoc tests following one-way ANOVA.


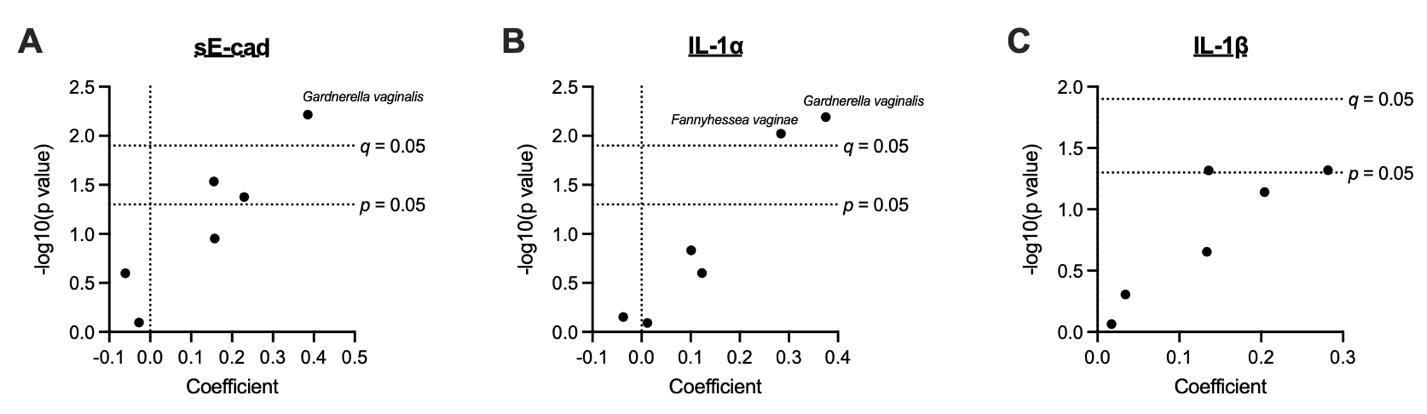


**Figure S2: BV-associated bacteria drive the association between total bacterial load and immune factors within CST-III.** Association between bacterial estimated absolute abundances and A) sE-cad, B) IL-1α, and C) IL-1β within CST-III. *P* values were corrected for multiple comparisons with the false discovery rate. Names of bacterial taxa that were significantly associated with an immune factor after correcting for multiple comparisons were annotated. Statistical tests were performed with MaAsLin2.


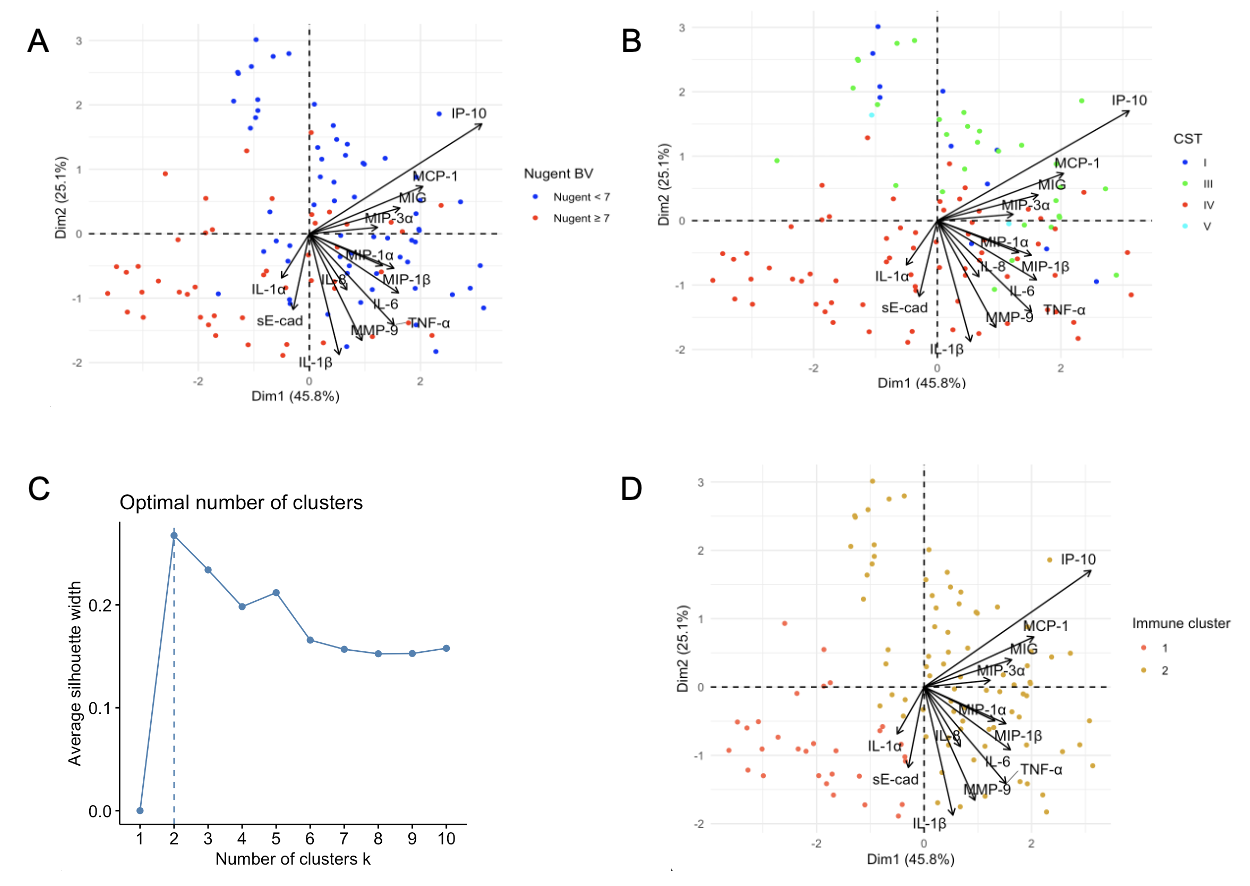


**Supplemental figure S3: Genital immune milieu cluster tightly with vaginal microbiota composition.** Principal component analysis (PCA) plots generated with soluble immune factor data from participants who had full immune data available, with participants labelled according to A) Nugent score, B) CST, and D) immune cluster identified with PAM clustering. Length of arrows correspond to individual variable (i.e., immune factor) contribution to PCA. C) Silhouette analysis plot indicating the optimal number of clusters for PAM clustering.

**
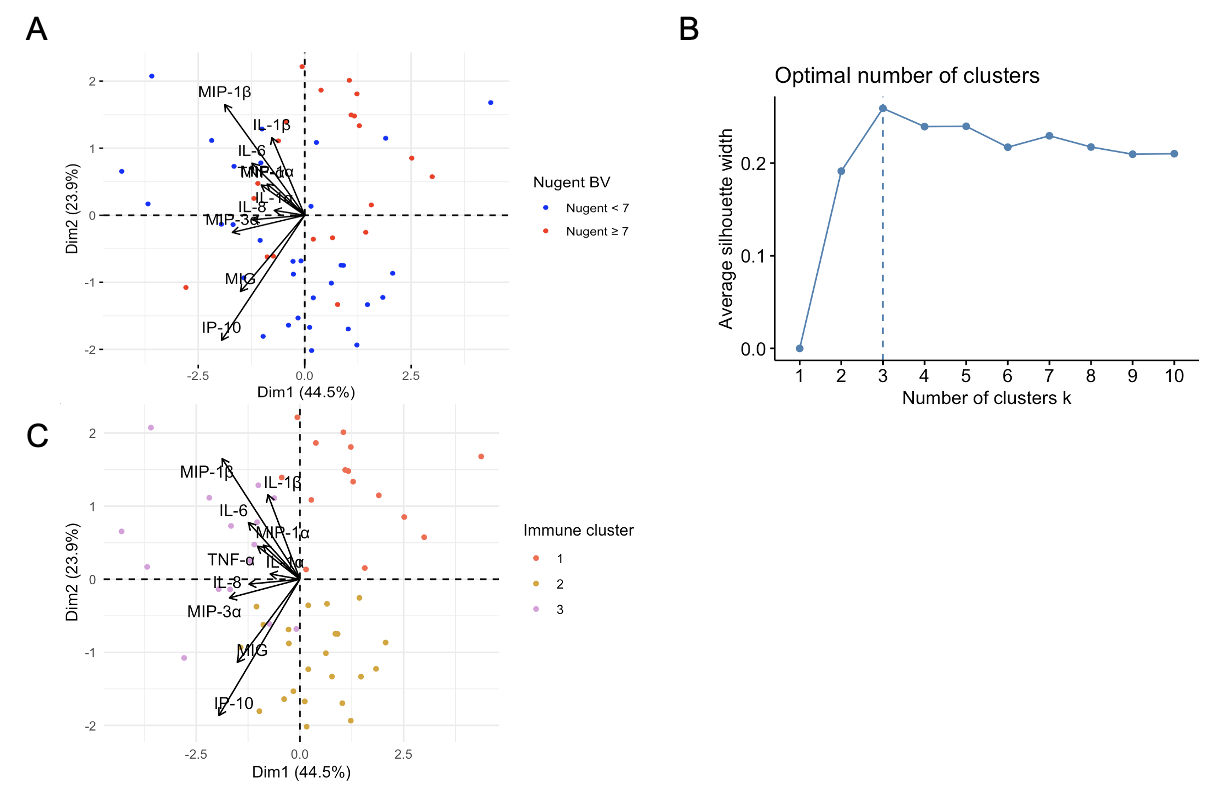
**

**Supplemental figure S4: Genital immune milieu is closely tied to vaginal microbiota composition in an independent, Uganda-based confirmatory cohort.** PCA plots generated with soluble immune factor data from participants who had full immune, bacterial load, and Nugent score data available, with participants labelled according to A) Nugent score, C) immune cluster identified with PAM clustering. Length of arrows correspond to individual variable (i.e., immune factor) contribution to PCA. B) Silhouette analysis plot indicating the optimal number of clusters for PAM clustering.

**Table S1: Association between vaginal CST and sociodemographic variables.**

| **Dependent variable** | **CST-I**  **(n = 19)** | **CST-II**  **(n = 1)** | **CST-III**  **(n = 50)** | **CST-IV**  **(n = 123)** | **CST-V**  **(n = 3)** | ***P* value** |
| --- | --- | --- | --- | --- | --- | --- |
| Age | 32  (20-45) | 26  (26-26) | 29.5  (20-45) | 30  (19-45) | 39  (22-41) | 0.748 |
| Contraceptive implant | 7  (37%) | 0  (0%) | 5  (10%) | 21  (17%) | 1  (33%) | 0.1044 |
| Injectable contraceptive | 2  (11%) | 1  (100%) | 10  (20%) | 19  (15%) | 1  (33%) | 0.1592 |
| IUD | 1  (5%) | 0  (0%) | 0  (0%) | 4  (3%) | 0  (0%) | 0.6956 |
| Oral contraceptive | 2  (11%) | 0  (0%) | 9  (18%) | 14  (11%) | 0  (0%) | 0.7148 |
| HSV-2 positive | 8  (42%) | 0  (0%) | 28  (56%) | 68  (55%) | 2  (67%) | 0.8947 |
| Douching | 10  (53%) | 1  (100%) | 24  (48%) | 57  (46%) | 2  (67%) | 0.7723 |
| Days since last vaginal sex | 2  (1-4) | 3  (3-3) | 3  (0-7) | 3  (1-7) | 3  (2-4) | 0.814 |
| Number of clients in past seven days | 3  (0-10) | 2  (2-2) | 3  (0-10) | 3  (0-10) | 0  (0-0) | 0.305 |
| Casual clients | 18  (95%) | 1  (100%) | 40  (80%) | 108  (88%) | 2  (67%) | 0.3829 |
| Regular clients | 19  (100%) | 1  (100%) | 44  (88%) | 117  (95%) | 3  (100%) | 0.3045 |
| Non-paying sex partner | 14  (74%) | 1  (100%) | 34  (68%) | 77  (63%) | 2  (67%) | 0.7944 |
| Descriptive statistics are median (range) or number (%). *P* values obtained with one-way ANOVA (if dependent variable is continuous) or Chi-Square test (if dependent variable is categorical). | | | | | | |

**Table S3: Nugent scores of women misclassified by the logistic regression model predicting Nugent BV with bacterial load in the SWOP cohort.**

|  | **Nugent score** | | | | | | | |  |
| --- | --- | --- | --- | --- | --- | --- | --- | --- | --- |
|  | 0 | 1 | 4 | 5 | 6 | 7 | 8 | 9 | **Total** |
| False negative | 0 (0%) | 0 (0%) | 0 (0%) | 0 (0%) | 0 (0%) | 10 (53%) | 8 (42%) | 1 (5%) | 19 |
| False positive | 1 (6%) | 1 (6%) | 6 (33%) | 2 (11%) | 8 (44%) | 0 (0%) | 0 (0%) | 0 (0%) | 18 |
| Nugent scores of false positives and false negatives expressed as a count and proportion relative to the total number of false positives or false negatives, respectively. | | | | | | | | | |

**Table S4: CST subgroups of women misclassified by the logistic regression model predicting molecular BV with bacterial load in the SWOP cohort.**

|  | **CST subgroups** | | | | | | | |
| --- | --- | --- | --- | --- | --- | --- | --- | --- |
|  | I-A | III-A | III-B | IV-B | IV-C1 | IV-C4 | V | **Total** |
| False negative | 0 (0%) | 0 (0%) | 0 (0%) | 9 (64%) | 4 (29%) | 1 (7%) | 0 (0%) | 14 |
| False positive | 5 (18%) | 15 (54%) | 8 (29%) | 0 (0%) | 0 (0%) | 0 (0%) | 1 (4%) | 28 |
| CST subgroups of false positives and false negatives expressed as a count and proportion relative to the total number of false positives or false negatives, respectively. | | | | | | | | |

**Table S5: Comparison of linear regression models predicting soluble immune factors with different vaginal microbiota characterization metrics.**

| **Immune factor** | **Predictor** | **Estimate** | **Adjusted R^2^** | ***P* value** | ***P* value (compared to bacterial load model)** |
| --- | --- | --- | --- | --- | --- |
| sE-cad | Bacterial load | 0.49496 | 0.285 | < 0.0001 |  |
|  | Nugent BV (ref: BV-positive) | 0.60906 | 0.288 | < 0.0001 | 0.483 |
|  | CST (ref: CST-I) |  | 0.461 | < 0.0001 | 0.006 |
|  | CST-III | 0.44388 |  | 0.0002 |  |
|  | CST-IV | 1.08331 |  | < 0.0001 |  |
|  | CST-V | 0.39986 |  | 0.1232 |  |
|  | Bacterial load + Nugent BV |  | 0.349 | < 0.0001 | 0.015 |
|  | Bacterial load | 0.30093 |  | < 0.0001 |  |
|  | Nugent BV (ref: BV-positive) | 0.37622 |  | < 0.0001 |  |
|  | Bacterial load + CST (ref: CST-I) |  | 0.508 | < 0.0001 | < 0.001 |
|  | Bacterial load | 0.23812 |  | < 0.0001 |  |
|  | CST-III | 0.40396 |  | 0.0003 |  |
|  | CST-IV | 0.90173 |  | < 0.0001 |  |
|  | CST-V | 0.41377 |  | 0.0952 |  |
|  | Nugent BV (ref: BV-positive) + CST (ref: CST-I) |  | 0.481 | < 0.0001 | 0.002 |
|  | Nugent BV | 0.22246 |  | 0.0051 |  |
|  | CST-III | 0.42937 |  | 0.0002 |  |
|  | CST-IV | 0.92605 |  | < 0.0001 |  |
|  | CST-V | 0.39986 |  | 0.1161 |  |
| IL-1α | Bacterial load | 0.43206 | 0.333 | < 0.0001 |  |
|  | Nugent BV (ref: BV-positive) | 0.53724 | 0.337 | < 0.0001 | 0.478 |
|  | CST (ref: CST-I) |  | 0.251 | < 0.0001 | 0.251 |
|  | CST-II | 0.30827 |  | 0.447 |  |
|  | CST-III | 0.09063 |  | 0.395 |  |
|  | CST-IV | 0.53120 |  | < 0.0001 |  |
|  | CST-V | -0.14821 |  | 0.546 |  |
|  | Bacterial load + Nugent BV (ref: BV-positive) |  | 0.409 | < 0.0001 | 0.015 |
|  | Bacterial load | 0.26231 |  | < 0.0001 |  |
|  | Nugent BV (ref: BV-positive) | 0.33340 |  | < 0.0001 |  |
|  | Bacterial load + CST (ref: CST-I) |  | 0.398 | < 0.0001 | 0.027 |
|  | Bacterial load | 0.32457 |  | < 0.0001 |  |
|  | CST-II | 0.38005 |  | 0.2968 |  |
|  | CST-III | 0.03769 |  | 0.6942 |  |
|  | CST-IV | 0.30847 |  | 0.0013 |  |
|  | CST-V | -0.12061 |  | 0.5840 |  |
|  | Nugent BV (ref: BV-positive) + CST (ref: CST-I) |  | 0.365 | < 0.0001 | 0.287 |
|  | Nugent BV | 0.39710 |  | < 0.0001 |  |
|  | CST-II | 0.30827 |  | 0.4094 |  |
|  | CST-III | 0.05886 |  | 0.5493 |  |
|  | CST-IV | 0.26269 |  | 0.0111 |  |
|  | CST-V | -0.14821 |  | 0.5123 |  |
| IP-10 | Bacterial load | -1.1347 | 0.295 | < 0.0001 |  |
|  | Nugent BV (ref: BV-positive) | -1.44953 | 0.365 | < 0.0001 | 0.129 |
|  | CST (ref: CST-I) |  | 0.300 | < 0.0001 | 0.386 |
|  | CST-II | -0.67106 |  | 0.513 |  |
|  | CST-III | 0.08188 |  | 0.762 |  |
|  | CST-IV | -1.32553 |  | < 0.0001 |  |
|  | CST-V | -0.07061 |  | 0.909 |  |
|  | Bacterial load + Nugent BV (ref: BV-positive) |  | 0.401 | < 0.0001 | 0.004 |
|  | Bacterial load | -0.5450 |  | 0.0005 |  |
|  | Nugent BV (ref: BV-positive) | -1.0400 |  | < 0.0001 |  |
|  | Bacterial load + CST (ref: CST-I) |  | 0.384 | < 0.0001 | 0.002 |
|  | Bacterial load | -0.7316 |  | < 0.0001 |  |
|  | CST-II | -0.8329 |  | 0.3874 |  |
|  | CST-III | 0.1978 |  | 0.4373 |  |
|  | CST-IV | -0.7739 |  | 0.0028 |  |
|  | CST-V | -0.1328 |  | 0.81972 |  |
|  | Nugent BV (ref: BV-positive) + CST (ref: CST-I) |  | 0.408 | < 0.0001 | 0.016 |
|  | Nugent BV | -1.01814 |  | < 0.0001 |  |
|  | CST-II | -0.67106 |  | 0.4768 |  |
|  | CST-III | 0.16499 |  | 0.5073 |  |
|  | CST-IV | -0.61196 |  | 0.0184 |  |
|  | CST-V | -0.07061 |  | 0.9015 |  |
| Regression estimates, R^2^ values, and *p* values obtained with linear regression. Rows display independent variables for each linear regression model for the prediction of the corresponding soluble immune factor. All models for each individual immune factor were compared to the model that included only bacterial load as an independent variable with ANOVA. | | | | | |

**Table S6: Comparison of sociodemographic characteristics based on availability of complete immune data in the SWOP cohort.**

| **Dependent variable** | **Complete immune data**  **(n=111)** | **Incomplete immune data**  **(n=85)** | ***P* value** |
| --- | --- | --- | --- |
| Age | 31 (20-45) | 29 (19-45) | 0.08617 |
| Contraceptive implant | 20 (18%) | 14 (16%) | 0.9257 |
| Injectable contraceptive | 18 (16%) | 15 (18%) | 0.942 |
| IUD | 3 (3%) | 2 (2%) | 1.000 |
| Oral contraceptive | 15 (14%) | 10 (12%) | 0.8826 |
| HSV-2 positive | 58 (52%) | 48 (56%) | 0.8143 |
| Douching | 63 (57%) | 39 (46%) | 0.1719 |
| Days since last vaginal sex | 2 (0-7) | 3 (1-7) | 0.4938 |
| Number of clients in past seven days | 3 (0-10) | 3 (0-10) | 0.8652 |
| Casual clients | 98 (88%) | 71 (84%) | 0.4539 |
| Regular clients | 104 (94%) | 80 (94%) | 1.000 |
| Non-paying sex partner | 75 (68%) | 53 (62%) | 0.5427 |
| Nugent BV | 47 (42%) | 43 (51%) | 0.3156 |
| Molecular BV (CST-IV) | 68 (61%) | 55 (65%) | 0.7299 |
| Descriptive statistics are median (range) or number (%). *P* values obtained with Mann-Whitney U test (if dependent variable is continuous) or Chi-Square test (if dependent variable is categorical). | | | |

**Table S7: Association between PAM immune cluster and sociodemographic variables.**

| **Dependent variable** | **PAM cluster #1**  **(n = 30)** | **PAM cluster #2**  **(n = 81)** | ***P* value** |
| --- | --- | --- | --- |
| Age | 30  (21-43) | 32  (20-45) | 0.4269 |
| Contraceptive implant | 5  (17%) | 15  (19%) | 1.000 |
| Injectable contraceptive | 5  (17%) | 13  (16%) | 1.000 |
| IUD | 1  (3%) | 2  (2%) | 1.000 |
| Oral contraceptive | 7  (23%) | 8  (10%) | 0.1262 |
| HSV-2 positive | 19  (63%) | 39  (48%) | 0.3253 |
| Syphilis positive | 0  (0%) | 2  (2%) | 0.565 |
| Douching | 16  (53%) | 47  (58%) | 0.8201 |
| Days since last vaginal sex | 3  (1-7) | 2  (0-5) | 0.4565 |
| Number of clients in past seven days | 3  (0-10) | 3  (0-10) | 0.3522 |
| Casual clients | 27  (90%) | 71  (88%) | 0.9928 |
| Regular clients | 30  (100%) | 74  (91%) | 0.221 |
| Non-paying sex partner | 20  (67%) | 55  (68%) | 1.000 |
| Descriptive statistics are median (range) or number (%). *P* values obtained with Mann-Whitney U test (if dependent variable is continuous) or Chi-square test (if dependent variable is categorical). | | | |

**Table S8: Sociodemographic factors for Uganda-based confirmatory cohort. N = 61.**

| **Variable** | **Median or number**  **(range or %)** |
| --- | --- |
| Age | 26 (18-43) |
| Contraceptive implant | 4 (7%) |
| Injectable contraceptive | 8 (13%) |
| Oral contraceptive | 1 (2%) |
| HSV-2 positive | 26 (43%) |
| Chlamydia positive | 2 (3%) |
| Gonorrhoea positive | 0 (0%) |
| *Trichomonas vaginalis* positive | 0 (0%) |
| Data are median (range) or number (%). | |

**Table S9: Nugent scores of women misclassified by the logistic regression model predicting Nugent BV with bacterial load in the Uganda-based confirmatory cohort.**

|  | **Nugent score** | | | |
| --- | --- | --- | --- | --- |
|  | 0 | 8 | 10 | **Total** |
| False negative | 0 (0%) | 5 (71%) | 2 (29%) | 7 |
| False positive | 4 (100%) | 0 (0%) | 0 (0%) | 4 |
| Nugent scores of false positives and false negatives expressed as a count and proportion relative to the total number of false positives or false negatives, respectively. | | | | |

**Table S10: Comparison of linear regression models predicting soluble immune factors with different vaginal microbiota characterization metrics in the confirmatory Uganda-based confirmatory cohort.**

| **Immune factor** | **Predictor** | **Estimate** | **Adjusted R^2^** | ***P* value** | ***P* value (compared to bacterial load model)** |
| --- | --- | --- | --- | --- | --- |
| IL-1α | Bacterial load | 0.2849 | 0.081 | 0.022 |  |
|  | Nugent BV (ref: BV-positive) | 0.3358 | 0.037 | 0.09065 | 0.303 |
|  | Bacterial load + Nugent BV |  | 0.070 | 0.06041 | 0.372 |
|  | Bacterial load | 0.2382 |  | 0.0972 |  |
|  | Nugent BV (ref: BV-positive) | 0.1444 |  | 0.5189 |  |
| IP-10 | Bacterial load | -0.5261 | 0.128 | 0.005 |  |
|  | Nugent BV (ref: BV-positive) | -1.1325 | 0.255 | < 0.0001 | 0.167 |
|  | Bacterial load + Nugent BV |  | 0.259 | 0.0002 | 0.088 |
|  | Bacterial load | -0.2164 |  | 0.26531 |  |
|  | Nugent BV (ref: BV-positive) | -0.9586 |  | 0.00264 |  |
| Regression estimates, R^2^ values, and *p* values obtained with linear regression. Rows display independent variables for each linear regression model for the prediction of the corresponding soluble immune factor. All models for each individual immune factor were compared to the model that included only bacterial load as an independent variable with ANOVA. | | | | | |

**Table S11: Primer and probe sequences for qPCR assays quantifying total bacterial load.**

| **Target** | **F primer (5' 🡪 3')** | **R primer (5' 🡪 3')** | **Probe (5' 🡪 3')** |
| --- | --- | --- | --- |
| 16S (total bacterial load) | TCC TAC GGG AGG CAG CAG T | GGA CTA CCA GGG TAT CTA ATC CTG TT | [FAM] CGT ATT ACC [ZEN] GCG GCT GCT GGC AC [IABkFQ] |
